# The social cost of carbon in regions and industries from ESG perspective - a case study of eight economic regions in China

**DOI:** 10.1186/s13021-026-00404-w

**Published:** 2026-02-07

**Authors:** Zihao Tian, Lixin Tian, Yixiang Zhao

**Affiliations:** 1 Business School, Hohai University, Nanjing, 211100 Jiangsu P.R. China; 2 Research Institute of Carbon Neutralization Development, Jiangsu University, Zhenjiang, 212013 Jiangsu P.R. China; 3 Jiangsu Province Engineering Research Center of Spatial Big Data, Nanjing Normal University, Nanjing, 210023 Jiangsu P.R. China

**Keywords:** Social cost of carbon, Carbon emission, Cooperative game, Non-cooperative games, Key industries, ESG

## Abstract

As a core metric for climate policy, the scientific estimation of carbon social costs is crucial for formulating mitigation strategies. However, traditional integrated assessment models predominantly focus on the global aggregate, failing to adequately account for regional heterogeneity, sectoral characteristics, and strategic interactions between regions. They also lack systematic integration of ESG principles. To address this, this paper examines regional and sectoral carbon social costs driven by ESG development. Through cooperative and non-cooperative games, we improve the integrated economic-environmental-climate development model, take the eight economic regions in China as an example, get the carbon social cost of each economic region and typical important industries, and obtain the key parameters and the evolution law of carbon social cost. The model categorizes the carbon emissions after the implementation of emission reduction policies under the ESG perspective into direct and indirect emissions. It studies the economic impacts of the two types of emissions before and after the implementation of emission reduction policies, and conducts research on the top four typical important industries (industry, construction, transportation, and power) that rank among the top four global CO_2_ emitters, to obtain the analytical solution of the social cost of carbon in the region and the typical important industries. In addition, this paper numerically simulates the social cost of carbon for the four industries under the baseline scenario, cooperative game scenario, non-cooperative game scenario, and temperature limitation scenario. The study shows that the social cost of carbon in the northern, southern and eastern coastal economic regions is higher than that in other economic regions, the social cost of carbon in the industrial and electric power industries in each economic region is higher than that in the building and transportation industries, and the more stringent the temperature limit is, the higher the social cost of carbon is in the economic regions.

## Introduction

Climate change and its impacts on socio-economic systems have become a focal point of concern for society as a whole, and the social cost of carbon (SCC), as a core measure of climate policy, is used as a guide for carbon pricing in carbon emissions regulation and assessment [1]. The social cost of carbon (SCC) is an expression of the economic cost of emitting an additional ton of carbon dioxide or its equivalent [2], which is defined as the marginal rate of substitution between carbon dioxide emissions and GDP [3]. In 2010, the Interagency Working Group (IWG) of the Federal government of the United States pioneered the use of the SCC to monetize the external costs of carbon emissions and to evaluate climate and energy regulations [4]. Estimating the social cost of carbon is an extremely complex and systematic endeavor that involves all the impacts of emissions, the carbon cycle, climate change, and regional policymaking and coordination, and the integration of the relevant impacts into a scientifically based estimate of the social cost of carbon is essential for policymakers to develop climate mitigation strategies and measures.

In the research on SCC estimation, the traditional IAM model is one of the main models to achieve the cost-benefit analysis for calculating SCC by simulating climate change pathways. Based on this, Pindyck [5] proposed a method to estimate the average SCC to provide guidance for policies over a longer period of time. In a quantitative study, David [6] quantitatively estimated the social cost of carbon using two integrated assessment models (FUND and RICE). The carbon cycle model in the DICE (Dynamic Integrated Climate Economy) model serves as an important prerequisite for estimating the social cost of carbon, and Tian et al. [7] improves the structure of the carbon cycle to be a nonlinear relationship. Van den Bijgaart et al. [8] studied the box carbon cycle system in a continuous time state and approximated the carbon social cost using the marginal cost method. These studies provide theoretical support for this paper to construct a new model for scientific assessment of SCC.

As an effective policy to adjust regional carbon emissions, the scientific and rational design of carbon tax will reduce the risk of climate change and minimize the cost of emission reduction. In the research on carbon tax, Golosov et al. [9] used the dynamic stochastic general equilibrium (DSGE) model with externalities to obtain the equations of marginal external damages on emissions and the optimal carbon tax. Rezai and Van der Ploeg [10] derived a simple rule for an almost optimal carbon tax. Withagen [11] evaluated the SCC simple rule is evaluated and explores the performance and robustness of the simple rule. Traeger [12] and Bretschger and Karydas [13] develop a model which provides closed solutions. These studies provide ideas for integrating the social cost of carbon and constructing an optimal carbon tax in a closed form.

With the deepening understanding of climate change, the study of the economic impact of damage caused by climate change has been a hot direction. As a long-term and uncertain public problem, scholars have achieved fruitful results by modeling climate change and its impacts on socio-economic systems. Gerlagh and Liski [14] investigated the future optimal pricing of the social cost of carbon under the uncertainty of climate change impacts. Cai and Lontzek [15] developed a nine-dimensional dynamic optimization model and discussed the social cost of carbon under various climate abrupt change scenarios. Van den Bremer and Van der Ploeg [16] adjusted the costs according to climate and economic risks and derived approximate tractable expressions. Bretschger and Pattakou [17] analyzed the impacts of climate change on the social cost of carbon and economic growth, and found that the setting of the damage function has a significant impact on the social cost of carbon. Russell et al. [18] investigated the RCP emission scenarios by constructing a simple carbon-climate model, and found that the estimation of the SCC depends on the specific damage function.

Emission reduction policy, as a macro policy to coordinate the green development of economic system and climate system, has been attracting much attention. In the study of emission reduction policies, Van der Ploeg and Rezai [19] derive simple formulas for the social cost of carbon and optimal emission reduction policies. Hassler and Krusell [20] develop a model integrating the climate and the global economy - the integrated assessment model, but the model only deals with fossil fuel trading and there are no markets across regions. Van der Ploeg and de Zeeuw [21] study the impact of productivity shocks due to climate change in cooperative and non-cooperative settings, with non-cooperative responses leading to more precautionary savings and shifting of carbon emission taxes. Duan et al. [22] multi-model study not only clarified that carbon intensity must decrease by over 60% under China’s 1.5 °C pathway and highlighted the critical role of CCS, but also revealed significant model differences in the social cost of carbon (SCC). Therefore, constructing an analytical framework for SCC that includes elements of regional games has become a direction that current research needs to focus on breaking through.

Inequality and disequilibrium between regions significantly affect the social cost of carbon. Hillebrand and Hillebrand [23] developed a dynamic general equilibrium model with an arbitrary number of different regions and derived an optimal climate policy consisting of an emissions tax and a transfer policy. Tol [24] found that the national social cost of carbon is much lower than the global social cost of carbon. Nordhaus [25] derives a characterization that emphasizes international non-cooperation in climate change mitigation and proposes climate clubs. Kornek et al. [26] calculates for the first time SCCs with heterogeneity between and within countries, proposes an optimal tax model for the social cost of carbon, explains inequalities between and within countries, and analyzes a more realistic heterogeneous regional carbon tax. Wang et al. [27] explores the impact of social economic factors and climate change on the social cost of carbon, and constructed an integrated framework for carbon emissions considering socioeconomic and climate factors, which includes shared socio-economic pathways (SSPs) and representative concentration pathways (RCPs).

**Fig. 1:**
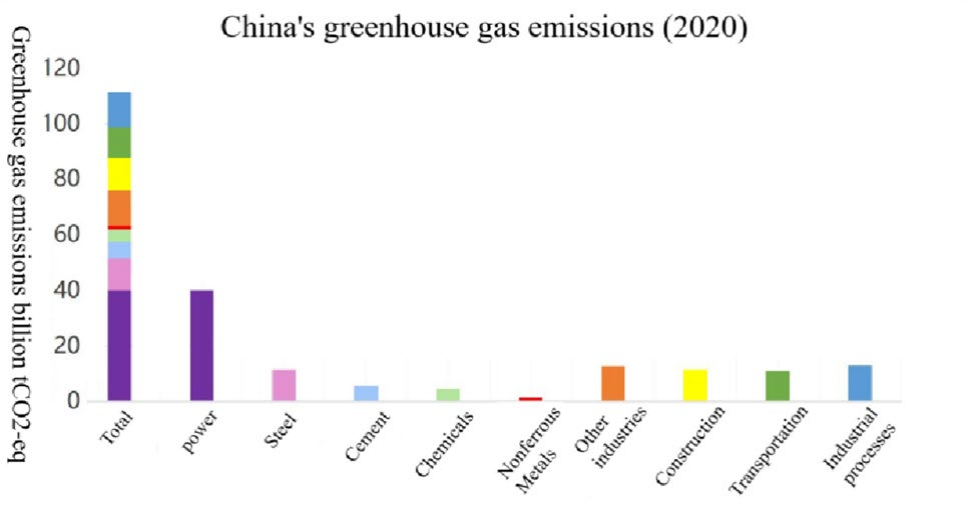
China’s greenhouse gas emissions in 2020

**Fig. 2:**
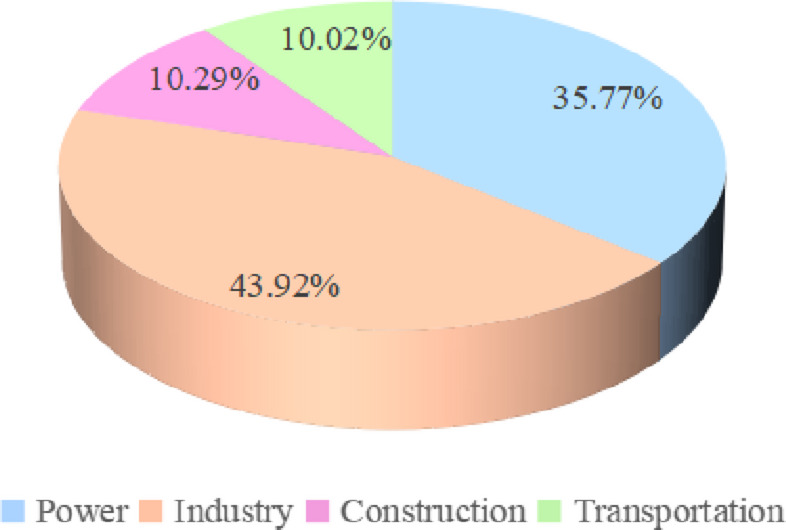
Percentage of greenhouse gas emissions in key industries

Carbon dioxide is the main cause of climate change, and carbon emissions in the narrow sense of the term mainly refer to carbon dioxide emissions. According to the CO_2_ Emissions Report 2022 released by the International Energy Agency on March 2, 2023, the power, industry, transportation and construction sectors account for the vast majority of global CO_2_ emissions. As a large carbon emitting country, China plays a key role in global governance. Many scholars have studied China’s carbon emissions and carbon emissions by sector. Chai Qimin et al. [28] studied the total energy consumption and structure of the three major sectors of industry, construction, and transportation during the 13th Five-Year Plan period. Chen et al. [29] studied as the four pillars of energy consumption industry, construction, transportation, and agricultural sectors accounted for 88.22% of China’s total carbon emissions in 2017. Xu Wei et al. [30] concluded that in 2019 China’s construction operation phase emissions were about 2.1 billion tCO_2_, accounting for about 20% of the total national carbon emissions. Li Xiaoyi et al. [31] concluded that CO_2_ emissions from the transportation sector accounted for about 11% of China’s total social CO_2_ emissions in 2019. Wang Lijuan et al. [32] pointed out that the power industry is the largest carbon emission sector in China, with carbon emissions accounting for more than 40% of the total national carbon emissions. Ni Bin [33] considered carbon emissions by industry, in which the power industry contributes 43% of carbon emissions, and the carbon emissions of the industry, construction, and transportation sectors account for 38%, 10%, and 9%. According to Zhang Xingfang et al. [34], CO_2_ emissions from industry are 35%, CO_2_ emissions from constructions are 6%, CO_2_ emissions from transportation are 10%, and CO_2_ emissions from power are 40%. The carbon emissions from industry, construction, transportation and power together account for about 91% of China’s total emissions. Based on Yan Gang et al. [35], China’s CO_2_ emissions from industry, construction, transportation, and power industry peaked in 2024, 2029, 2028, and 2031, respectively. Zhang Jing et al. [36] studied that China’s total carbon emission reduction in key industries and fields by 2030 was 34.7 × 108 t, and the four fields of power, industry, transportation, and construction accounted for 72.1%, 15.1%, and 15.1%, respectively. 72.1%, 15.1%, 3.1% and 9.7% respectively. According to Wei Yiming et al. [37], the emissions of CO_2_ in greenhouse gases in China in 2020 were approximately 11.2 billion tons. The specific industry emissions are shown in Fig. 1, and the CO_2_ emissions from the four sectors are shown in Fig. 2. Among them, the CO_2_ emissions from the power sector account for 35.77%; the industrial sector accounts for 43.92%; the construction sector accounts for 10.29%, and the transportation sector accounts for 10.02%.

ESG stands for “Environmental, Social, and Governance”, and the sustainable development of the global economy and society requires the practice of ESG principles, which have been developed for 17 years since they were formally proposed in 2004. Countries around the world continue to promote the coordinated development of environment, society, and governance in accordance with ESG principles. Zefeng Lv and Qingxue Ma [38] argue that companies need to control greenhouse gas emissions and resource utilization on the environmental side, safeguard the rights and interests of employees and communities on the social side, and establish a transparent management structure on the governance side. Yu Qian and Yichao Liu [39] explore the impacts of the impact of ESG on carbon emission efficiency and concluded that ESG is particularly significant in non-polluting and non-state-owned enterprises in improving the carbon emission efficiency of companies. Yamin Xie [40] analyzed the relationship between ESG performance and sustainable growth of listed companies in China, and used a natural language processing method of deep learning to find that ESG has a significant positive impact on sustainable growth, and that carbon sentiment has a significant role in enhancing ESG performance plays an important role in enhancing the role of ESG performance on sustainable growth. Alessio Baratta et al. [41] found that the combination of environmental and governance innovations and socially sustainable policies has a significant positive effect on reducing industrial carbon emissions.

To summarize, scholars both domestically and internationally predominantly employ comprehensive assessment models to study the social cost of carbon, deriving general formulas for this metric. However, these studies are primarily applicable at the global economic level and remain insufficient in addressing interregional trade interactions, the systematic integration of ESG principles, and considerations of industry heterogeneity. Notably, there is a lack of interactive research incorporating game theory methodologies. The following questions are therefore key to the study:

Question: How to obtain the social cost of carbon for each region and industry based on ESG principles, regional and industry characteristics?

This paper constructs regional and industry carbon social cost models under the ESG concept driven by climate change impacts. Using cooperative and non-cooperative game theory, do the model to analyze and answer the above questions. Obtain the analytical solution of carbon social cost for each economic region and key industry, analyze the impact of key variables, and quantify the impact of trade on carbon social cost.

To derive an analytical solution for the Social Cost of Carbon (SCC) and thereby clearly reveal the underlying mechanisms by which ESG factors, regional trade, and strategic behavior influence it, this study incorporates two key assumptions in the modeling process: (1) The capital accumulation equation is a linear differential equation; (2) The immediate output damage from temperature increases is temporarily excluded from the production function, setting ${D_{\mathrm{n}}}(T_{t}^{{at}})=1$. These simplifications inevitably introduce corresponding limitations.

First, the assumption of linear capital accumulation (as shown in Eq. (23)) offers the advantage of transforming a complex nonlinear dynamic system into a manageable form, enabling us to obtain closed-form solutions for SCC, the optimal consumption rate, and the emission control rate. However, this assumption ignores the potential existence of diminishing returns on capital, which may lead to an overestimation of the long-term capital accumulation rate and consequently affect the valuation of SCC in the distant future.

Second, the omission of direct temperature-induced damage to output represents a key distinction from classical integrated assessment models like DICE. This approach prevents the model from capturing direct shocks to economic fundamentals through channels such as agriculture, health, and labor productivity, potentially leading to an underestimation of overall SCC levels.

Nevertheless, we contend that the findings derived under these assumptions retain significant theoretical reference value and policy implications for the following reasons:

(1) Core mechanisms are preserved: This study does not aim to provide an absolutely precise point estimate of SCC, but rather focuses on analyzing the relative differences in regional and sectoral SCC driven by ESG factors and their interaction mechanisms. The model fully retains the three core mechanisms driving SCC: ① Converting emissions into temperature rise through the nonlinear carbon cycle and temperature system; ② Temperature directly erodes capital stock through the temperature term $- {\xi _n}T_{t}^{{at}}{K_{nt}}$ in the capital accumulation equation (as shown in Eq. (3)), representing climate damage to infrastructure, production equipment, and other capital assets; ③ Regions mutually internalize this capital damage through game-theoretic behavior and trade linkages. Thus, the core economic cost of climate change—the destruction of wealth stock—is fully accounted for.

(2) Robustness of Findings: Our key conclusions—such as “SCC is lower in cooperative games than non-cooperative games,” “regional trade is a crucial damage transmission channel,” and “higher SCC in industrial and power sectors”—primarily rely on the model’s structural design (e.g., game type, trade network, sector heterogeneity) and the incorporation of the ESG factors. These structural elements constitute the model’s core innovation, and the resulting conclusions exhibit robustness to specific assumptions regarding functional forms.

(3) Laying Groundwork for Future Research: The analytical solution derived in this study provides a valuable benchmark model. It clearly demonstrates the direction of marginal effects of various parameters (e.g., climate sensitivity, time preference, trade weights) on SCC, offering explicit theoretical expectations for subsequent research. Future work can build upon this framework by incorporating more complex nonlinear damage functions and capital dynamics, employing numerical methods for solution to validate and calibrate the robustness of this paper’s conclusions, and pursuing higher-precision quantitative assessments.

In summary, this model successfully reveals the complex dynamics determining regional and sectoral carbon social costs from an ESG perspective through controlled simplification. Its findings provide robust theoretical support for understanding the intrinsic drivers of cross-regional climate policy cooperation and formulating differentiated emission reduction strategies.

The highlights of this paper and the differences with previous studies are as follows: (1) Distinguishing from the traditional path of aggregation estimation based on the IAM model of [5–8], this paper constructs a new carbon social cost assessment framework that integrates ESG principles (environment, society, governance), regional differences and industry characteristics, takes green governance inputs and other factors into account, and targets the four major power, industry, building and transportation A differentiated assessment model is established for the four key areas, and a nonlinear relationship between carbon social cost and emission control rate is found. (2) Different from the game model of [19–21], which does not consider inter-regional interaction, this paper applies cooperative and non-cooperative game methods, and systematically analyzes the dynamic influence mechanism of regional trade on carbon social cost under the framework of ESG concept and carbon neutrality target. By constructing a multi-subject game model, the carbon social cost reduction effect brought by regional cooperation is quantified, and key policy parameters such as optimal carbon emission rate and optimal consumption are derived, which makes up for the theoretical defects of the traditional research that ignores the strategic interaction between regions. (3) Compared with [23–27], which focuses on the differences at the national level but lacks industry scenario simulation, this paper analyzes the carbon social cost practiced by eight economic regions and industries in China through scenario simulation, and analyzes the evolution of key parameters under different temperature rise backgrounds, which breaks through the limitations of the traditional macro-aggregation analysis, and provides a scientific basis for the formulation of differentiated regional climate policies.

The paper is organized as follows: Sect. 1: introduction; Sect. 2: construction of a new economic, climate and utility model; Sect. 3: non-cooperative game scenarios; Sect. 4: cooperative game scenarios; Sect. 5: numerical simulations and evolutionary analyses; and Sect. 6: conclusion.

## Improved governance, environmental, and social modeling and utility construction

**Fig. 3:**
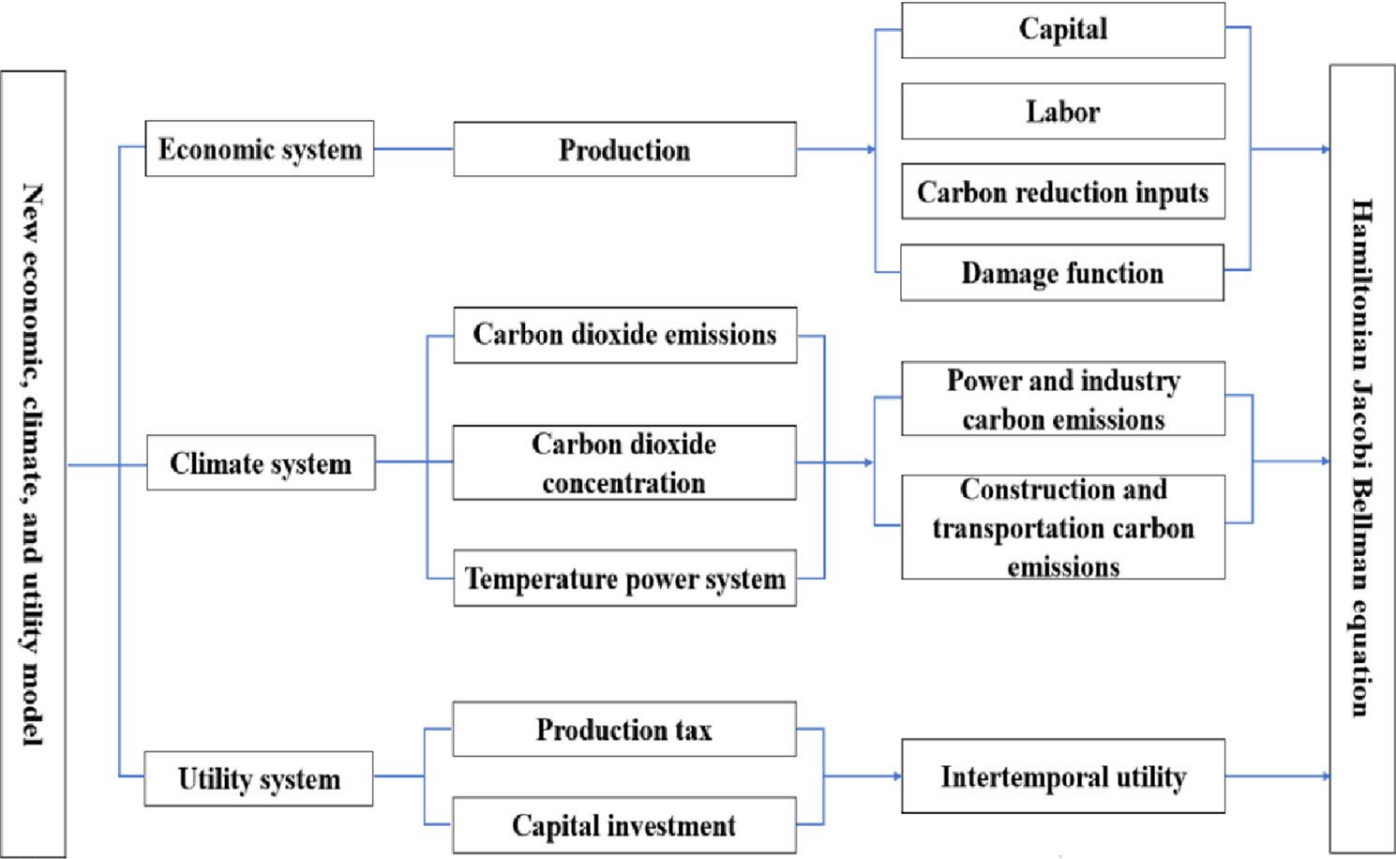
A Research Framework for Economic, Climate, and Utility Modeling under the Novel ESG Dimension

In this paper, an improved production function is constructed in the economic system under the ESG perspective through the economic inputs to reduce carbon emissions under green and low-carbon governance, subdividing the key industries such as industry, construction, transportation, and power. The carbon emissions considered in the carbon system come from the, industry, construction, transportation and power key industries. A new social utility function incorporating the total inputs of the total products consumed to reduce carbon emissions is constructed in the utility system. The research framework of the model is shown in Fig. 3.

This study operationalizes the core dimensions of ESG principles and integrates them into a model framework. Its operational pathways and theoretical contributions are as follows:

Environmental Dimension: Primarily manifested in production and investment. Within the production function, ${e_n}(t)$ represents dedicated green capital investments (e.g., new energy technologies, energy efficiency enhancements) made to reduce carbon emissions. Its output share $1 - {\alpha _n} - {\beta _n}$ reflects the substitution of green factors for traditional capital. This dimension lowers the Social Cost of Carbon (SCC) from the supply side by enhancing green total factor productivity and reducing carbon intensity.

Social Dimension: Primarily manifested in consumption. Within the utility function, we introduce the $carb$ term, representing the additional cost or voluntary payment borne by residents for green products and low-carbon services (i.e., consumption generating indirect emission reductions). The parameter $\gamma$ adjusts the weighting of traditional consumption versus green consumption in the utility. This dimension influences SCC from the demand side by shaping social preferences and consumption structures, reflecting the pull effect of heightened public environmental awareness on emission reductions.

Governance Dimension: Primarily manifested in public expenditure. The climate mitigation expenditures$A_{n}^{{ind}} (t),A_{n}^{{arct}} (t),A_{n}^{{trans}} (t),A_{n}^{{pow}} (t)$ across sectors in the model essentially quantify government and corporate governance actions—such as purchasing emission reduction technologies, implementing regulatory measures, and constructing infrastructure. The emission control rate ${\mu _{ni}}$ serves as a key indicator of governance efficiency. This dimension directly enhances emission reduction efficiency by optimizing public resource allocation and institutional design, thereby lowering SCC from the policy side.

In summary, the marginal innovation of this model lies in systematically integrating the three ESG dimensions—often treated separately in prior research—within a unified dynamic general equilibrium framework, while clarifying their respective transmission pathways affecting carbon social costs: the environmental dimension through technological innovation, the social dimension through preference guidance, and the governance dimension through policy intervention. This transforms SCC estimation from a purely climate economics issue into a multidimensional decision problem integrating environmental responsibility, social consensus, and governance effectiveness.

### Economic-governance model

**Table 1:** Composition of the eight major economic regions

Economic Regions	Regional composition (by province)
Northeast Economic Region (Northeast E-Z)	Liaoning, Jilin, Heilongjiang
Eastern Coastal Economic Region (Eastern C-E-Z)	Shanghai, Jiangsu, Zhejiang
Northern Coastal Economic Region (Northern C-E-Z)	Beijing, Tianjin, Hebei, Shandong
Southern Coastal Economic Region (Southern C-E-Z)	Fujian, Guangdong, Hainan
Middle reaches of the Yellow River economic Region (M-R Yellow E-Z)	Shanxi, Inner Mongolia, Henan, Shaanxi
Middle reaches of the Yangtze River Economic Region (M-R Yangtze E-Z)	Anhui, Jiangxi, Hunan, Hubei
Southwest Economic Region (Southwest E-Z)	Guangxi, Chongqing, Sichuan, Guizhou, Yunnan
Northwest Economic Region (Northwest E-Z)	Gansu, Qinghai, Ningxia, Xinjiang

In this paper, on the one hand, based on the ESG perspective, we consider that residents practice green behaviors actively, consciously, and self-discipline, and that green behavioral choice preferences become the general norm. On the other hand, China is divided into eight economic regions, see Table 1. Due to the heterogeneity of the economic regions, the production of each economic region varies. Therefore, in this paper, in the Cobb-Douglas (D-S) production function, factors such as capital, labor and capital inputs to reduce carbon emission reduction are considered, and the impact of damage on output is considered. Thus, we obtain the production function for the economic Regions.

1 $${Y_{nt}}={A_n}\left( t \right){K_n}{\left( t \right)^{{\alpha _n}}}{L_n}{\left( t \right)^{{\beta _n}}}{e_n}{\left( t \right)^{1 - {\alpha _n} - {\beta _n}}}{D_n}\left( {T_{t}^{{at}}} \right).$$

Among them, ${A_n}\left( t \right)$ is the total factor productivity (TFP) of the economic Region; ${K_n}\left( t \right)$ is capital; ${L_n}\left( t \right)$ is the labor force used for production in the economic Region; ${e_n}\left( t \right)$ represents the capital invested in reducing carbon emissions in the production of products under the requirements of green and low-carbon economic governance; ${\alpha _n}$ is the output share of capital; ${\beta _n}$ is the output share of labor force; $1 - {\alpha _n} - {\beta _n}$ represents the output share of capital input to reduce carbon emissions, and ${D_n}\left( {T_{t}^{{at}}} \right)$ is a damage function.

Based on the perfectly competitive market assumption, output is equilibrated with aggregate consumption, aggregate capital investment and climate change mitigation expenditures. i.e.

2 $$ \begin{aligned} Y_{{nt}} =\; & C_{n} (t) + \underbrace {{{\mathrm{I}}_{n}^{1} (t) + {\mathrm{I}}_{n}^{e} (t)}}_{{{\text{Total capital investment}}}} \\ & + \underbrace {{A_{n}^{{ind}} (t) + A_{n}^{{arct}} (t) + A_{n}^{{trans}} (t) + A_{n}^{{pow}} (t)}}_{{{\text{Total mitigation expenditures}}}}. \\ \end{aligned} $$

Among them, ${\mathrm{I}}_{n}^{1}(t)$ is the capital invested in the base without taking into account the policy and other factors, and ${\rm I}_{n}^{e}(t)$ is the capital invested in the economy to reduce carbon emissions. ${\rm I}_{n}^{e}(t)$ can be understood as the economic region is willing to invest in emission reduction projects to reduce the amount of carbon dioxide accumulated in order to pursue a good environment, which is different from the corresponding capital invested in the ${e_n}\left( t \right)$ of the whole company as mentioned above. This means that the economic region receives a positive externality in terms of environmental improvement from technological innovations, environmental quality products and new energy products, and pays to internalize the positive environmental externality in order to eliminate it. $A_{n}^{{ind}}(t)$, $A_{n}^{{arct}}(t)$, $A_{n}^{{trans}}(t)$, $A_{n}^{{pow}}(t)$ indicates expenditures on climate change mitigation in key sectors in the economic region: industry, construction, transportation, and power. This model assumes all markets operate under perfect competition and clear instantly. Beyond the production function in Eq. (1) and the output equilibrium constraint in Eq. (2), it implicitly incorporates a crucial market clearing condition: the aggregate supply of final and intermediate goods across all economic regions equals total demand.

Climate change has an impact on the growth rate of capital. When the influence of temperature on capital change is introduced into the capital accumulation equation, the equation of motion of capital stock is:

3 $$\mathop {{K_{nt}}}\limits^{.} =L_{n}^{1}(t)+L_{n}^{e}(t) - \left( {\delta _{n}^{k}+{\xi _n}T_{t}^{{at}}} \right){K_{nt}}.$$

Among them, the parameter $\delta _{n}^{k}$represents the depreciation rate of capital and ${\xi _n}$ is the damage parameter of the economic Region. The last item on the right side of Eq. (3) represents the impact of capital depreciation and climate damage on capital.

### Climate-environment modeling

This paper does not consider non-CO_2_ greenhouse gases, but only considers the four main sources of CO_2_ emissions from human activities: industry, construction, transportation, and power Industry includes steel, cement, chemicals, other key industries, and industrial processes. This paper only considers the carbon emissions and exogenous sources of key industries such as industry, construction, transportation, and power in each period $t$. Exogenous sources come from biological processes in China, including the recording of CO_2_ generated by deforestation and land use. The total emissions are given by these five parts,

4 $${E_{nt}}=E_{{nt}}^{{{\mathrm{ind}}}}+E_{{nt}}^{{{\mathrm{arct}}}}+E_{{nt}}^{{{\mathrm{trans}}}}+E_{{nt}}^{{{\mathrm{pow}}}}+E_{{nt}}^{{{\mathrm{land}}}}.$$

The emission control rates of the industry, construction, transportation, and power industries in the economic Region $n$ are ${\mu _{n1}}$,${\mu _{n2}}$,${\mu _{n3}}$,${\mu _{n4}}$. respectively. The emission control rate is defined as the proportion of reducing carbon dioxide emissions, which can reduce current CO_2_ emissions and suppress the rise of CO_2_ concentration in the atmosphere. If $0 \leqslant {\mu _{n1}},{\mu _{n2}},{\mu _{n3}},{\mu _{n4}} \leqslant 1$ is satisfied, these industries can reduce CO_2_ emissions based on their emission control rates, and the CO_2_ emissions from industry, construction, transportation, and power in the economic Region can be expressed as:

5 $$\begin{gathered} E_{{nt}}^{{ind}} (t) = \left( {Y_{{nt}} - carb} \right)\sigma _{n}^{1} (t)\left( {1 - \mu _{{n1}} (t)} \right), \hfill \\ E_{{nt}}^{{{\mathrm{arct}}}} (t) = \left( {Y_{{nt}} - carb} \right)\sigma _{n}^{2} (t)\left( {1 - \mu _{{n2}} (t)} \right), \hfill \\ E_{{nt}}^{{trans}} (t) = \left( {Y_{{nt}} - carb} \right)\sigma _{n}^{3} (t)\left( {1 - \mu _{{n3}} (t)} \right), \hfill \\ E_{{nt}}^{{pow}} (t) = \left( {Y_{{nt}} - carb} \right)\sigma _{n}^{4} (t)\left( {1 - \mu _{{n4}} (t)} \right). \hfill \\ \end{gathered}$$

**Fig. 4:**
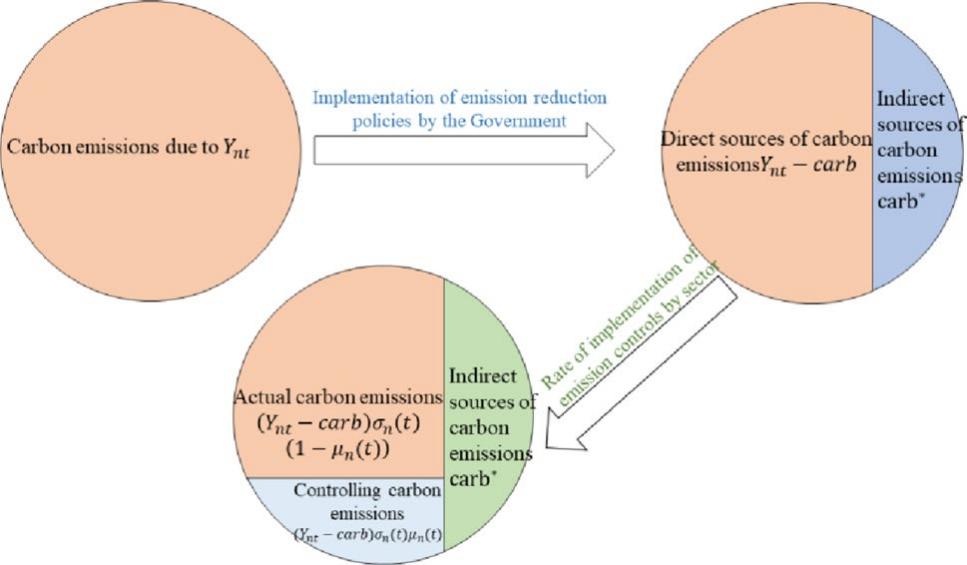
Sources of carbon emissions before and after the policy

Among them, $carb$ represents the economic inputs that individuals need to bear to reduce carbon emissions in an economic region, including capital expenditures for emission reductions as well as the consumption costs of internalizing the positive externalities of environmental improvements generated by technological innovation goods, environmental quality goods, and new energy goods. In the DICE model, carbon emissions are set to be proportional to output. This paper plots the division of carbon emissions into two categories after the implementation of emission reduction policies: (1) direct carbon emissions: carbon emissions from production activities that originate from ${Y_{nt}} - carb$; (2) indirect carbon emissions: whose economic source is $carb$, but $carb$ itself does not produce carbon emissions. That is, the portion of carbon emissions that is reduced after the implementation of the emission reduction policy. (3) Considering the prominence of emissions from important carbon-emitting industries, it is necessary to implement carbon-emission control for each industry in order to be effective in reducing emissions. As in Fig. 4. $\sigma _{n}^{1}(t)$,$\sigma _{n}^{2}(t)$,$\sigma _{n}^{3}(t)$,$\sigma _{n}^{4}(t)$, respectively, represent the carbon intensity of the industrial, construction, transportation, and power output in the economic Region before emission reduction. Carbon intensity reflects how many emissions need to be released to produce one unit of output. It will decrease over time due to external technological advancements. Indeed, the indirect source of carbon emissions $carb$ in Fig. 4 is due to the economic input capital ${\rm I}_{n}^{e}(t)$ in Eq. (2) that reduces carbon emissions, and the control of carbon emission $\left( {{Y_{nt}} - carb} \right){\sigma _n}(t){\mu _n}(t)$ is due to the expenditures on climate change mitigation by industries in the economic region $A_{n}^{{ind}}(t)$,$A_{n}^{{arct}}(t)$,$A_{n}^{{trans}}(t)$,$A_{n}^{{pow}}(t)$ are obtained.

Therefore, the national CO_2_ emissions are,

6 $${E_t}=\sum\limits_{{n=1}}^{N} {\left[ {E_{n}^{{\operatorname{in} d}}(t)+E_{n}^{{arct}}(t)+E_{n}^{{trans}}(t)+E_{n}^{{pow}}(t)+E_{n}^{{land}}(t)} \right]} .$$

Following Nordhaus [24], the mitigation expenditures for industry, construction, transportation, and power are:

7 $$\begin{gathered} A_{n}^{{ind}}(t)={a_n}(t){\mu _{n1}}{(t)^{{b_n}}}{Y_{nt}},A_{n}^{{arct}}(t)={a_n}(t){\mu _{n2}}{(t)^{{b_n}}}{Y_{nt}}, \hfill \\ A_{n}^{{trans}}(t)={a_n}(t){\mu _{n3}}{(t)^{{b_n}}}{Y_{nt}},A_{n}^{{pow}}(t)={a_n}(t){\mu _{n4}}{(t)^{{b_n}}}{Y_{nt}}. \hfill \\ \end{gathered}$$

Among them, ${b_n}>1$. Mitigation expenditure is a convex function of emission control rate. ${a_n}(t)$ is a function of expenditures to reduce carbon emissions, which decreases with technological progress.

In addition, in the carbon cycle module, the three-layer atmosphere-ocean carbon cycle framework of Nordhaus [42] is adopted and the nonlinear carbon emission response mechanism proposed by Tian et al. [7] is introduced, while the temperature system is based on the classical temperature dynamics equations of Nordhaus [43] combined with the assumption of linear radiative forcing of Christoph Hambel et al. [44].

### Utility modeling of regional economic systems

From Fig. 4, after the implementation of emission reduction policy, carbon emission is composed of direct carbon emission and indirect carbon emission. At this time, the utility function considers the corresponding consumption and the input capital $carb$ that generates indirect carbon emissions, at this time the carb is the indirect carbon emission source $car{b^*}$and the expenditure of controlling carbon emissions $\left( {{Y_{nt}} - carb} \right){\sigma _n}(t){\mu _n}(t)$. Then the utility function of introducing economic region $n$ is

8 $${U_n}\left( {{C_{nt}},{L_{nt}},carb} \right)={L_{nt}}\left[ {\frac{{{{\left( {\frac{{\gamma {C_{nt}}+(1 - \gamma )carb}}{{{L_{nt}}}}} \right)}^{1 - {\eta _n}}}}}{{1 - {\eta _n}}}} \right].$$

Among them, ${U_n}$ is the instantaneous utility of economic Region $n$; ${C_n}$ and ${L_n}$ are the consumption of common goods and total consumption population of economic Region $n$; ${\eta _n}$ is the Marginal utility elasticity of consumption, which reflects the substitutability of intergenerational consumption. The larger the ${\eta _n}$, the lower the substitutability of intergenerational consumption.

Consider the special situation of ${\eta _n}=1$:

9 $$\mathop {\lim }\limits_{{{\eta _n} \to 1}} {U_n}\left( {{C_{nt}},{L_{nt}},carb} \right)={L_{nt}}\ln \left( {\frac{{\gamma {C_{nt}}+(1 - \gamma )carb}}{{{L_{nt}}}}} \right).$$

According to Hu et al. [45] improvement of the utility function with constant elasticity of substitution (CES) utility function, the consumption bundle is written as:

10 $${C_{nt}}={\left( {\sum\limits_{{l=1}}^{N} {\beta _{l}^{n}{{\left( {C_{{lt}}^{n}} \right)}^{{\rho _n}}}} } \right)^{{1 \mathord{\left/ {\vphantom {1 {{\rho _n}}}} \right. \kern-0pt} {{\rho _n}}}}}.$$

Among them, $C_{l}^{n}$ represents the consumption of goods produced by the economic Region $l$ through trade. $\beta _{l}^{n}$ represents the preference selection coefficient of consumer goods, while ${\rho _n}<1$ and ${\varsigma _n}=\frac{1}{{1 - {\rho _n}}}$ represent the substitution elasticity between goods in economic Region. The preference selection coefficient satisfies $\sum\nolimits_{{l=1}}^{N} {\beta _{l}^{n}=1}$ for all $n=1, \ldots ,N.$

Consider the situation of ${\rho _n}=0$:

11 $$\begin{gathered} \mathop {\lim }\limits_{{{\rho _n} \to 0}} \ln {C_{nt}}=\ln {\left( {C_{{1t}}^{n}} \right)^{\beta _{1}^{n}}}{\left( {C_{{2t}}^{n}} \right)^{\beta _{2}^{n}}} \cdots {\left( {C_{{Nt}}^{n}} \right)^{\beta _{N}^{n}}}, \hfill \\ {C_{nt}}={\left( {C_{{1t}}^{n}} \right)^{\beta _{1}^{n}}}{\left( {C_{{2t}}^{n}} \right)^{\beta _{2}^{n}}} \cdots {\left( {C_{{Nt}}^{n}} \right)^{\beta _{N}^{n}}}=\prod\limits_{{l=1}}^{N} {{{\left( {C_{{lt}}^{n}} \right)}^{\beta _{l}^{n}}}} . \hfill \\ \end{gathered}$$

It is important for consumers to maximize not only current utility but also intertemporal utility, that is:

12 $$\int\limits_{0}^{\infty } {\left\{ {\left( {L_{{nt}} \frac{{\left( {\gamma C_{{nt}} + (1 - \gamma )carb} \right)^{{1 - \eta _{n} }} L_{{nt}} ^{{\eta _{n} - 1}} }}{{1 - \eta _{n} }}} \right)\exp ( - \delta _{n} t)} \right\}dt} .$$

Among them, ${\delta _n}>0$ represents the subjective time Bank rate (the time preference rate selected between current consumption and future consumption). When ${\delta _n}$ decreases, it indicates a smaller difference between current and future consumption, and when ${\delta _n}$ increases, it indicates a greater preference for current consumption.

## SCC under non-cooperative games between economic regions

### Social cost of carbon under the implementation of optimal carbon tax

Under the implementation of the emission reduction policy, the emissions from the industrial, construction, transportation, and power sectors in the economic region $\:n$ under the implementation of the emission control rate are respectively:

13 $$\begin{gathered} E_{{nt}}^{{ind}}(t)=\left( {{Y_{nt}} - carb} \right)\sigma _{n}^{1}(t)\left( {1 - {\mu _{n1}}(t)} \right)=E_{{nt}}^{1}(t)\left( {1 - {\mu _{n1}}(t)} \right), \hfill \\ E_{{nt}}^{{{\mathrm{arct}}}}(t)=\left( {{Y_{nt}} - carb} \right)\sigma _{n}^{2}(t)\left( {1 - {\mu _{n2}}(t)} \right)=E_{{nt}}^{2}(t)\left( {1 - {\mu _{n2}}(t)} \right), \hfill \\ E_{{nt}}^{{trans}}(t)=\left( {{Y_{nt}} - carb} \right)\sigma _{n}^{3}(t)\left( {1 - {\mu _{n3}}(t)} \right)=E_{{nt}}^{3}(t)\left( {1 - {\mu _{n3}}(t)} \right), \hfill \\ E_{{nt}}^{{pow}}(t)=\left( {{Y_{nt}} - carb} \right)\sigma _{n}^{4}(t)\left( {1 - {\mu _{n4}}(t)} \right)=E_{{nt}}^{4}(t)\left( {1 - {\mu _{n4}}(t)} \right). \hfill \\ \end{gathered}$$

Among them, $E_{{nt}}^{1}(t)$,$E_{{nt}}^{2}(t)$,$E_{{nt}}^{3}(t)$,$E_{{nt}}^{4}(t)$ denote the carbon emissions of the four sectors, namely industry, construction, transportation and power, respectively, when the emission control rate is not implemented. When the industry emits one ton of carbon dioxide $\Delta E_{{nt}}^{{ind}}(t)=1tC$, the output is $\Delta {Y_{nt}}=\frac{1}{{\sigma _{n}^{1}(t)\left( {1 - \omega {\chi _n}} \right)\left( {1 - {\mu _{n1}}(t)} \right)}}$, and the industrial mitigation expenditure is $A_{n}^{{ind}}(t)={a_n}(t){\mu _{n1}}{(t)^{{b_n}}}{Y_{nt}}$, then the marginal cost of the industry to avoid this ton of carbon dioxide is:

14 $$\begin{aligned} MRA_{n}^{{ind}} (t) & = a_{n} (t)b_{n} \mu _{{n1}} (t)^{{b_{n} - 1}} \Delta Y_{{nt}} \\ & = \mu _{{n1}} (t)^{{b_{n} - 1}} \frac{{a(t)b_{n} }}{{\sigma _{n}^{1} (t)\left( {{\text{1 - }}\omega \chi _{n} } \right)\left( {1 - \mu _{{n1}} (t)} \right)}}. \\ \end{aligned}$$

Similarly, the marginal cost of avoiding one ton of carbon dioxide in the construction, transportation, and power industries is:

15 $$\begin{gathered} MRA_{n}^{{arct}}(t)={\mu _{n2}}{(t)^{{b_n} - 1}}\frac{{a(t){b_n}}}{{\sigma _{n}^{2}(t)\left( {{\mathrm{1-}}\omega {\chi _n}} \right)\left( {1 - {\mu _{n2}}(t)} \right)}}, \hfill \\ MRA_{n}^{{trans}}(t)={\mu _{n3}}{(t)^{{b_n} - 1}}\frac{{a(t){b_n}}}{{\sigma _{n}^{3}(t)\left( {{\mathrm{1-}}\omega {\chi _n}} \right)\left( {1 - {\mu _{n3}}(t)} \right)}}, \hfill \\ MRA_{n}^{{pow}}(t)={\mu _{n4}}{(t)^{{b_n} - 1}}\frac{{a(t){b_n}}}{{\sigma _{n}^{4}(t)\left( {{\mathrm{1-}}\omega {\chi _n}} \right)\left( {1 - {\mu _{n4}}(t)} \right)}}. \hfill \\ \end{gathered}$$

The social cost of carbon is the marginal damage caused by the extra emission of one ton of carbon dioxide. In the optimal state, marginal damage equals to marginal cost. That is, the social cost of carbon is equal to the optimal carbon tax. In the optimal state, the social cost of carbon of industry, construction, transportation and power industry is:

16 $$\begin{gathered} SC{C_{nt}}(ind)=\tau _{{nt}}^{*}\left( {ind} \right)=\mu _{{n1}}^{*}{(t)^{{b_n} - 1}}\frac{{a(t){b_n}}}{{\sigma _{n}^{1}(t)\left( {{\mathrm{1-}}\omega {\chi _n}} \right)\left( {1 - \mu _{{n1}}^{*}(t)} \right)}}, \hfill \\ SC{C_{nt}}(arct)=\tau _{{nt}}^{*}\left( {arct} \right)=\mu _{{n2}}^{*}{(t)^{{b_n} - 1}}\frac{{a(t){b_n}}}{{\sigma _{n}^{2}(t)\left( {{\mathrm{1-}}\omega {\chi _n}} \right)\left( {1 - \mu _{{n2}}^{*}(t)} \right)}}, \hfill \\ SC{C_{nt}}(trans)=\tau _{{nt}}^{*}\left( {trans} \right)=\mu _{{n3}}^{*}{(t)^{{b_n} - 1}}\frac{{a(t){b_n}}}{{\sigma _{n}^{3}(t)\left( {{\mathrm{1-}}\omega {\chi _n}} \right)\left( {1 - \mu _{{n3}}^{*}(t)} \right)}}, \hfill \\ SC{C_{nt}}(pow)=\tau _{{nt}}^{*}\left( {pow} \right)=\mu _{{n4}}^{*}{(t)^{{b_n} - 1}}\frac{{a(t){b_n}}}{{\sigma _{n}^{4}(t)\left( {{\mathrm{1-}}\omega {\chi _n}} \right)\left( {1 - \mu _{{n4}}^{*}(t)} \right)}}. \hfill \\ \end{gathered}$$

Among them, $\mu _{{n1}}^{*}(t)$,$\mu _{{n2}}^{*}(t)$,$\mu _{{n3}}^{*}(t)$,$\mu _{{n4}}^{*}(t)$ represents the optimal emission control rate for the industrial, construction, transportation, and power industries in the economic Region $n$, and $\tau _{{nt}}^{*}$ represents the optimal carbon tax.

The above derivation establishes that, within a general equilibrium framework, the social cost of carbon (SCC) theoretically equals the marginal cost of avoiding the last ton of carbon emissions—that is, the optimal carbon tax. However, this equilibrium outcome is highly dependent on a critical premise: the existence of a global social planner capable of implementing optimal emission reduction strategies uniformly, disregarding interregional conflicts of interest. Real-world climate governance lacks such a central authority, with regions typically acting as rational agents making independent decisions based on self-interest maximization. This decentralization of decision-making power makes interregional strategic interactions the core mechanism shaping the final formation of SCC. Therefore, to assess carbon social costs more realistically, we must shift our research perspective from a single optimal control problem to strategic games among multiple heterogeneous regions.

### Social cost of carbon under the optimal emission reduction strategy in the economic region

At each time point $t \in [0,\infty )$, each economic Region will use a non-cooperative game to choose the optimal consumption reduction strategy. The consumption reduction strategy is recorded as $\pi =\left( {C_{1}^{n}, \ldots ,C_{N}^{n},\mu _{n}^{1},\mu _{n}^{2},\mu _{n}^{3},\mu _{n}^{4}} \right)_{{n=1}}^{N}.$Each economic Region gains utility from consumption bundle ${C_n}$, while the utility of economic Region $n$ under $\pi$ consumption reduction strategy is recorded as ${J_n}\left( \pi \right).$

Each economic Region maximizes its consumption utility by implementing consumption reduction strategies. Each economic Region predicts the actions of all other economic Regions and selects an acceptable consumption reduction strategy ${\pi _n}=\left( {C_{1}^{n}, \ldots ,C_{N}^{n},\mu _{n}^{1},\mu _{n}^{2},\mu _{n}^{3},\mu _{n}^{4}} \right)_{{n=1}}^{N}$ to maximize the utility ${J_n}\left( {{\pi _n}} \right)$ of this economic Region. If each economic Region has no motivation to deviate from its strategy, Nash equilibrium $\left( {\pi _{n}^{*}} \right)_{{n=1}}^{N}$ will be achieved. That is to say, the strategies $\left( {{\pi _n}|\pi _{{ - n}}^{*}} \right)=\left( {\pi _{1}^{*}, \ldots ,\pi _{{n - 1}}^{*},\pi _{n}^{*},\pi _{{n+1}}^{*}, \ldots ,\pi _{N}^{*}} \right)$ and $n=1, \ldots ,N.$ selected after economic Region $n$ responds to the optimal behavior of other economic Regions have ${J_n}\left( {{\pi _n}|\pi _{{ - n}}^{*}} \right) \leqslant {J_n}\left( {\pi _{n}^{*}} \right)$. The maximum value of utility obtained by economic Region $n$ is represented by ${J^n}$, and the value function ${J^n}$ can be expressed as ${J^n}=\mathop {\hbox{max} }\limits_{{{\pi _n}}} \left\{ {{J_n}\left( {{\pi _n}|\pi _{{ - n}}^{*}} \right)} \right\}.$

The optimal trajectory of the state variable generated by Nash equilibrium is represented as $\left( {{M^*},{T^*},{K^*}} \right).$ Among them, $M=\left( {{M^{at}},{M^{uo}},{M^{lo}}} \right)$ and$T=\left( {{T^{at}},{T^o}} \right).$

The optimal consumption reduction strategy can be determined by solving a coupled Hamilton-Jacobian Bellman (HJB) equation system. The HJB equation of economic Region $n\left( {n=1, \ldots ,N} \right)$ is expressed as:

17 $$\begin{aligned} \delta _{n} J^{n} = & \mathop {\max }\limits_{{\chi _{1}^{n} , \cdots ,\chi _{N}^{n} ,\mu _{{n(i = 1,2,3,4)}}^{i} }} \\ & \times \left\{ \begin{gathered} \frac{{\partial J^{n} }}{{\partial t}} + \delta _{n} \log \left( {\gamma C_{{nt}} + (1 - \gamma )carb} \right) \hfill \\ + \sum\limits_{{l = 1}}^{N} {\frac{{\partial J^{n} }}{{\partial K_{l} }}\frac{{\partial K_{l} }}{{\partial t}} + \frac{{\partial J^{n} }}{{\partial M^{{at}} }}\frac{{\partial M^{{at}} }}{{\partial t}} + \frac{{\partial J^{n} }}{{\partial M^{{uo}} }}\frac{{\partial M^{{uo}} }}{{\partial t}}} \hfill \\ + \frac{{\partial J^{n} }}{{\partial M^{{lo}} }}\frac{{\partial M^{{lo}} }}{{\partial t}} + \frac{{\partial J^{n} }}{{\partial T^{{at}} }}\frac{{\partial T^{{at}} }}{{\partial t}} + \frac{{\partial J^{n} }}{{\partial T^{o} }}\frac{{\partial T^{o} }}{{\partial t}} \hfill \\ \end{gathered} \right\}. \\ \end{aligned}$$

The purpose of this paper is to obtain the analytical solution of social cost of carbon, for which the capital accumulation equation is assumed to be the Linear differential equation of ${K_n}.$Therefore, in Eq. (1), we set ${\alpha _n}=1,{\beta _n}=0,{D_n}\left( {T_{t}^{{at}}} \right)=1$ without considering the impact of temperature on output. The consumption output ratio of $n$ economic Region is recorded as${\chi _n}=\frac{{{C_n}}}{{{Y_n}}}.$ For Eq. (3), we have,

18 $$\begin{gathered} d{K_{nt}}=\left[ {{\rm I}_{n}^{1}(t)+{\rm I}_{n}^{e}(t) - \delta _{n}^{k}{K_{nt}} - {\xi _n}T_{t}^{{at}}{K_{nt}}} \right]dt \hfill \\ ={K_{nt}}\left[ {{A_n}\left( {1 - a(t)\left( {\sum\limits_{{i=1}}^{4} {{\mu _{ni}}{{(t)}^{{b_n}}}} } \right) - {\chi _n}} \right) - \delta _{n}^{k} - {\xi _n}T_{t}^{{at}}} \right]dt. \hfill \\ \end{gathered}$$

Where ${g_n}$ can be written as Eq. (18), representing the total economic growth rate before the impact of climate change,

19 $${g_n}(t,{x_n},{\mu _{ni}})={A_n}\left( {1 - a(t)\left( {\sum\limits_{{i=1}}^{4} {{\mu _{ni}}{{(t)}^{{b_n}}}} } \right) - {\chi _n}} \right) - \delta _{n}^{k}.$$

The optimal change in output ${Y_n}$ is denoted as $\overline {{{Y_n}}}$, and the total emissions are rewritten as ${E_{nt}}=\sum\limits_{{i=1}}^{4} {\left( {\overline {{{Y_n}}} - carb} \right)\sigma _{n}^{i}(t)\left( {1 - \mu _{n}^{i}} \right)} +E_{n}^{{land}}.$ We have $\chi _{l}^{n}=\frac{{C_{l}^{n}}}{{Y_{l}^{n}}},$ which represents the proportion of trade consumption from the economic Region $l$ to the output of the economic Region $l$ in the economic Region $n$. Let $\frac{{{Y_k}}}{{{Y_n}}}=\omega _{n}^{k}$, then there is,

20 $$\begin{gathered} {C_{nt}}=C_{{nt}}^{n}+\sum\limits_{{k \ne n}} {C_{k}^{n}} =\prod\limits_{{l=1}}^{N} {{{\left( {\chi _{l}^{n}{A_l}{K_l}} \right)}^{\beta _{l}^{n}}}} , \hfill \\ \frac{{{C_n}}}{{{Y_n}}}=\frac{{C_{n}^{n}}}{{{Y_n}}}+\sum\limits_{{k \ne n}} {\frac{{\chi _{k}^{n}{Y_k}}}{{{Y_n}}}} =\frac{{C_{n}^{n}}}{{{Y_n}}}+\sum\limits_{{k \ne n}} {\chi _{k}^{n}\omega _{n}^{k}} , \hfill \\ {\chi _n}=\chi _{n}^{n}+\sum\limits_{{k \ne n}} {\chi _{k}^{n}\omega _{n}^{k}=} \chi _{n}^{n}\omega _{n}^{n}+\sum\limits_{{k \ne n}} {\chi _{k}^{n}\omega _{n}^{k}=} \sum\limits_{{k=1}}^{N} {\chi _{k}^{n}\omega _{n}^{k}} , \hfill \\ \end{gathered}$$

21 $${\delta _n}\log \left( {{C_{nt}}} \right)={\delta _n}\beta _{l}^{n}\sum\limits_{{l=1}}^{N} {\log \left( {\chi _{l}^{n}{A_l}{K_l}} \right)} .$$

Combined with the assumptions of the carbon cycle module, the above HJB equation is rewritten as

22 $$ \begin{aligned} \delta _{n} J^{n} = & \mathop {\max }\limits_{{\chi _{1}^{n} , \cdots ,\chi _{N}^{n} ,\mu _{{n(i = 1,2,3,4)}}^{i} }} \\ & \times \left\{ \begin{gathered} \frac{{\partial J^{n} }}{{\partial t}} + \delta _{n} \beta _{l}^{n} \sum\limits_{{l = 1}}^{N} {\log \left[ {\left( {\gamma + \left( {1 - \gamma } \right)\omega } \right)\chi _{l}^{n} A_{l} K_{l} } \right]} \hfill \\ + \sum\limits_{{l = 1}}^{N} {\frac{{\partial J^{n} }}{{\partial K_{l} }}K_{l} \left[ {g_{n} (t,\chi _{n} ,\mu _{{ni(i = 1,2,3,4)}} ) - \xi _{n} T_{t}^{{at}} } \right]} \hfill \\ + \frac{{\partial J^{n} }}{{\partial T^{{at}} }}\left( {\varepsilon _{1} \left[ {\eta _{0} + \eta _{1} \frac{{M_{t}^{{at}} }}{{M_{*}^{{at}} }} + F^{{ex}} (t)} \right] - \left( {\varepsilon _{2} + \varphi _{{21}} } \right)T^{{at}} + \varphi _{{21}} T^{o} } \right) \hfill \\ + \frac{{\partial J^{n} }}{{\partial T^{o} }}\left( {\varphi _{{12}} T^{{at}} - \varphi _{{12}} T^{o} } \right) \hfill \\ + \frac{{\partial J^{n} }}{{\partial M^{{at}} }}\left( { - \phi _{{12}} M^{{at}} + \phi _{{21}} M^{{uo}} + \sum\limits_{{l = 1}}^{N} {E_{l} \left( {1 - \frac{{\sum\limits_{{l = 1}}^{N} {E_{l} } }}{{zk_{e} }}} \right)} } \right) \hfill \\ + \frac{{\partial J^{n} }}{{\partial M^{{uo}} }}\left( {\phi _{{12}} M^{{at}} - \left( {\phi _{{21}} + \phi _{{23}} } \right)M^{{uo}} + \phi _{{32}} M^{{lo}} } \right) \hfill \\ + \frac{{\partial J^{n} }}{{\partial M^{{lo}} }}\left( {\phi _{{23}} M^{{uo}} - \phi _{{32}} M^{{lo}} } \right) \hfill \\ \end{gathered} \right\}. \\ \end{aligned} $$

To determine the value function ${J^n}$, we speculate that its form is:

23 $$\begin{aligned} J^{n} (t,M,T,K) = & \sum\limits_{{l = 1}}^{N} {\beta _{l}^{n} \log (K_{l} ) - P_{{M^{{at}} }}^{n} M^{{at}} } \\ & - P_{{M^{{uo}} }}^{n} M^{{uo}} - P_{{M^{{lo}} }}^{n} M^{{lo}} \\ & - P_{{T^{{at}} }}^{n} T^{{at}} - P_{{T^{o} }}^{n} T^{o} + p^{n} (t). \\ \end{aligned}$$

Substitute into Eq. (22), and for the convenience of calculation, consider the case of $K=1$, then,

24 $$ \begin{aligned} {\mathrm{0}} = & \mathop {\max }\limits_{{\chi _{1}^{n} , \cdots ,\chi _{N}^{n} ,\mu _{{n(i = 1,2,3,4)}}^{i} }} \\ & \times \left\{ \begin{gathered} p_{t}^{n} + \delta _{n} \beta _{l}^{n} \sum\limits_{{l = 1}}^{N} {\log \left[ {\left( {\gamma + \left( {1 - \gamma } \right)\omega } \right)\chi _{l}^{n} A_{l} K_{l} } \right]} \hfill \\ + \sum\limits_{{l = 1}}^{N} {\beta _{l}^{n} \left[ {g_{n} (t,\chi _{n} ,\mu _{{ni(i = 1,2,3,4)}} ) - \xi _{n} T_{t}^{{at}} } \right]} \hfill \\ - P_{{T^{{at}} }}^{n} \left( {\varepsilon _{1} \left[ {\eta _{0} + \eta _{1} \frac{{M_{t}^{{at}} }}{{M_{*}^{{at}} }} + F^{{ex}} (t)} \right] - \left( {\varepsilon _{2} + \varphi _{{21}} } \right)T^{{at}} + \varphi _{{21}} T^{o} } \right) \hfill \\ - P_{{T^{o} }}^{n} \left( {\varphi _{{12}} T^{{at}} - \varphi _{{12}} T^{o} } \right) - P_{{M^{{lo}} }}^{n} \left( {\phi _{{23}} M^{{uo}} - \phi _{{32}} M^{{lo}} } \right) \hfill \\ - P_{{M^{{at}} }}^{n} \left( { - \phi _{{12}} M^{{at}} + \phi _{{21}} M^{{uo}} + \sum\limits_{{l = 1}}^{N} {E_{l} \left( {1 - \frac{{\sum\limits_{{l = 1}}^{N} {E_{l} } }}{{zk_{e} }}} \right)} } \right) \hfill \\ - P_{{M^{{uo}} }}^{n} \left( {\phi _{{12}} M^{{at}} - \left( {\phi _{{21}} + \phi _{{23}} } \right)M^{{uo}} + \phi _{{32}} M^{{lo}} } \right) \hfill \\ + \delta _{n} \left( {P_{{M^{{at}} }}^{n} M^{{at}} + P_{{M^{{uo}} }}^{n} M^{{uo}} + P_{{M^{{lo}} }}^{n} M^{{lo}} + P_{{T^{{at}} }}^{n} T^{{at}} + P_{{T^{o} }}^{n} T^{o} + p^{n} (t)} \right) \hfill \\ \end{gathered} \right\}. \\ \end{aligned} $$

For the two-dimensional temperature system variable $\left( {{T^{at}},{T^o}} \right),$ it is necessary to meet:

25 $$\begin{aligned} - & \varphi _{{21}} T^{o} P_{{T^{{at}} }}^{n} + \varphi _{{12}} T^{o} P_{{T^{o} }}^{n} + \delta _{n} P_{{T^{o} }}^{n} T^{o} = 0, \\ & P_{{T^{o} }}^{n} = \frac{{\varphi _{{21}} }}{{\delta _{n} + \varphi _{{12}} }}P_{{T^{{at}} }}^{n} . \\ \end{aligned}$$

26 $$\begin{aligned} - & \sum\limits_{{l = 1}}^{N} {\beta _{l}^{n} } \xi _{l} T_{t}^{{at}} + \left( {\varepsilon _{2} + \varphi _{{21}} } \right)T^{{at}} P_{{T^{{at}} }}^{n} - \varphi _{{21}} T^{{at}} P_{{T^{o} }}^{n} + \delta _{n} P_{{T^{{at}} }}^{n} T^{{at}} = 0, \\ & \sum\limits_{{l = 1}}^{N} {\beta _{l}^{n} } \xi _{l} = \left( {\delta _{n} + \varepsilon _{2} + \varphi _{{21}} - \frac{{\varphi _{{12}} \varphi _{{21}} }}{{\delta _{n} + \varphi _{{12}} }}} \right)P_{{T^{{at}} }}^{n} , \\ & P_{{T^{{at}} }}^{n} = \frac{{\sum\limits_{{l = 1}}^{N} {\beta _{l}^{n} } \xi _{l} }}{{\delta _{n} + \varepsilon _{2} + \varphi _{{21}} - \frac{{\varphi _{{12}} \varphi _{{21}} }}{{\delta _{n} + \varphi _{{12}} }}}}. \\ \end{aligned}$$

For three-dimensional carbon system variables$\left( {{M^{at}},{M^{uo}},{M^{lo}}} \right)$, it is necessary to meet:

$$\left\{ {\begin{array}{*{20}{c}} {{\phi _{12}}P_{{{M^{at}}}}^{n}{M^{at}} - {\phi _{12}}P_{{{M^{uo}}}}^{n}{M^{at}} - {\varepsilon _1}{\eta _1}\frac{{M_{t}^{{at}}}}{{M_{*}^{{at}}}}P_{{{T^{at}}}}^{n}+{\delta _n}P_{{{M^{at}}}}^{n}{M^{at}}=0} \\ { - {\phi _{21}}P_{{{M^{at}}}}^{n}{M^{uo}}+\left( {{\phi _{21}}+{\phi _{23}}} \right)P_{{{M^{uo}}}}^{n}{M^{uo}} - {\phi _{23}}P_{{{M^{lo}}}}^{n}{M^{uo}}+{\delta _n}P_{{{M^{uo}}}}^{n}{M^{uo}}=0} \\ { - {\phi _{32}}P_{{{M^{uo}}}}^{n}{M^{lo}}+{\phi _{32}}P_{{{M^{lo}}}}^{n}{M^{lo}}+{\delta _n}P_{{{M^{lo}}}}^{n}{M^{lo}}=0} \end{array}} \right.$$

27 $$ \begin{aligned} \left( {\begin{array}{*{20}c} {P_{{M^{{at}} }}^{n} } \\ {P_{{M^{{uo}} }}^{n} } \\ {P_{{M^{{lo}} }}^{n} } \\ \end{array} } \right) = & \left( {\begin{array}{*{20}c} {\delta _{n} + \phi _{{12}} } & { - \phi _{{12}} } & 0 \\ { - \phi _{{12}} } & {\delta _{n} + \phi _{{21}} + \phi _{{23}} } & { - \phi _{{23}} } \\ 0 & { - \phi _{{32}} } & {\delta _{n} + \phi _{{32}} } \\ \end{array} } \right)^{{ - 1}} \\ & \times \left( {\begin{array}{*{20}c} {{{\varepsilon _{1} \eta _{1} P_{{T^{{at}} }}^{n} } \mathord{\left/ {\vphantom {{\varepsilon _{1} \eta _{1} P_{{T^{{at}} }}^{n} } {M_{*}^{{at}} }}} \right. \kern-\nulldelimiterspace} {M_{*}^{{at}} }}} \\ 0 \\ 0 \\ \end{array} } \right). \\ \end{aligned} $$

Noting that $${\left( {\begin{array}{*{20}{c}} {{\delta _n}+{\phi _{12}}}&{ - {\phi _{12}}}&0 \\ { - {\phi _{12}}}&{{\delta _n}+{\phi _{21}}+{\phi _{23}}}&{ - {\phi _{23}}} \\ 0&{ - {\phi _{32}}}&{{\delta _n}+{\phi _{32}}} \end{array}} \right)^{ - 1}}=N$$, ${N_{1,1}}$ denote the first row and first column elements of the matrix $N$, then,

28 $$P_{{{M^{at}}}}^{n}={N_{1,1}}\frac{{{\varepsilon _1}{\eta _1}P_{{{T^{at}}}}^{n}}}{{M_{*}^{{at}}}}={N_{1,1}}\frac{{{\varepsilon _1}{\eta _1}}}{{M_{*}^{{at}}}}\frac{{\sum\limits_{{l=1}}^{N} {\beta _{l}^{n}} {\xi _l}}}{{{\delta _n}+{\varepsilon _2}+{\varphi _{21}} - \frac{{{\varphi _{12}}{\varphi _{21}}}}{{{\delta _n}+{\varphi _{12}}}}}}.$$

The above Eq. (24) can be written as:

29 $$ \begin{aligned} 0 = & \mathop {\max }\limits_{{\chi _{1}^{n} , \cdots ,\chi _{N}^{n} ,\mu _{{n(i = 1,2,3,4)}}^{i} }} \\ & \times \left\{ \begin{gathered} p_{t}^{n} + \delta _{n} \sum\limits_{{l = 1}}^{N} {\beta _{l}^{n} \log \left[ {\left( {\gamma + \left( {1 - \gamma } \right)\omega } \right)\chi _{l}^{n} A_{l} } \right]} \hfill \\ + \sum\limits_{{l = 1}}^{N} {\beta _{l}^{n} g_{l} (t,\chi _{l} ,\mu _{{li(i = 1,2,3,4)}} )} \hfill \\ - P_{{M^{{at}} }}^{n} \sum\limits_{{l = 1}}^{N} {E_{l} \left( {1 - \frac{{\sum\limits_{{l = 1}}^{N} {E_{l} } }}{{zk_{e} }}} \right)} - P_{{T^{{at}} }}^{n} \varepsilon _{1} \left[ {\eta _{0} + F^{{ex}} (t)} \right] \hfill \\ + \delta _{n} \left( { - p^{n} (t)} \right) \hfill \\ \end{gathered} \right\}. \\ \end{aligned} $$

So, the simplified HJB equation is:

30 $$ \begin{aligned} \delta _{n} p^{n} (t) = & \mathop {\max }\limits_{{\chi _{1}^{n} , \cdots ,\chi _{N}^{n} ,\mu _{{n(i = 1,2,3,4)}}^{i} }} \\ & \times \left\{ \begin{gathered} p_{t}^{n} + \delta _{n} \sum\limits_{{l = 1}}^{N} {\beta _{l}^{n} \log \left[ {\left( {\gamma + \left( {1 - \gamma } \right)\omega } \right)\chi _{l}^{n} A_{l} } \right]} \hfill \\ + \sum\limits_{{l = 1}}^{N} {\beta _{l}^{n} g_{l} (t,\chi _{l} ,\mu _{{li(i = 1,2,3,4)}} )} \hfill \\ - P_{{M^{{at}} }}^{n} \sum\limits_{{l = 1}}^{N} {E_{l} \left( {1 - \frac{{\sum\limits_{{l = 1}}^{N} {E_{l} } }}{{zk_{e} }}} \right)} - \varepsilon _{1} \left[ {\eta _{0} + F^{{ex}} (t)} \right]P_{{T^{{at}} }}^{n} \hfill \\ \end{gathered} \right\}. \\ \end{aligned} $$

Then the first-order conditions for the emission control rates on the industrial, construction, transportation, and power industries are:

$$ \begin{aligned} \frac{{\partial \delta _{n} J^{n} }}{{\partial \mu _{{ni}} }} - \lambda _{{ni}} = & \frac{{\partial J^{n} }}{{\partial K_{l} }}K_{l} \frac{{\partial g_{n} (t,x_{n} ,y_{n} ,z_{n} ,\mu _{{ni}} )}}{{\partial \mu _{{ni}} }} \\ & - \frac{{\partial J^{n} }}{{\partial M^{{at}} }}E_{{nt}}^{i} (t)\overline{{Y_{n} }} \sigma _{n}^{i} (t) \\ & + \frac{{\partial J^{n} }}{{\partial M^{{at}} }}\frac{{2E_{{nt}}^{i} (t)}}{{zk_{e} }}\overline{{Y_{n} }} \sigma _{n}^{i} (t) - \lambda _{{ni}} = 0 \\ \end{aligned} $$

31 $$ \begin{aligned} \beta _{n}^{n} A_{n} \left( {a(t)b_{n} \mu _{{n1}} ^{{b_{n} - 1}} } \right) = & P_{{M^{{at}} }}^{n} E_{{nt}}^{i} (t) \\ & \times \left( {1 - \frac{{2\left( {\sum\limits_{{i = 1}}^{4} {E_{{nt}}^{i} (t)\left( {1 - \mu _{{ni}} (t)} \right)} } \right)}}{{zk_{e} }}} \right) - \lambda _{{ni}} . \\ \end{aligned} $$

The first order condition for consumption rate is:

32 $$\begin{gathered} \frac{{\partial {J^n}}}{{\partial {K_n}}}{K_n}\frac{{\partial {g_n}(t,{\chi _n},{\mu _{ni(i=1,2,3,4)}})}}{{\partial {x_n}}}\omega _{l}^{n}= - \frac{{{\delta _n}\beta _{l}^{n}}}{{\chi _{l}^{n}}}, \hfill \\ \beta _{n}^{n}{A_n}\omega _{l}^{n}\chi _{l}^{n}={\delta _n}\beta _{l}^{n}. \hfill \\ \end{gathered}$$

Since${\chi _n}=\sum\nolimits_{{k=1}}^{N} {\chi _{k}^{n}\omega _{n}^{k}} ,$ its summation yields $\beta _{n}^{n}{A_n}=\frac{{{\delta _n}}}{{{\chi _n}}}.$Because of ${b_n}>1,$ this paper only considers ${b_n}=2,$ the optimal emission control rate for industry, construction, transportation, and power in the economic Region $n$ is:

$$ \begin{aligned} \mu _{{ni}} = & \frac{{zk_{e} \left( {P_{{M^{{at}} }}^{n} E_{{nt}}^{i} (t) - \lambda _{{ni}} } \right) - 2P_{{M^{{at}} }}^{n} \left[ {E_{{nt}}^{i} (t)} \right]^{2} }}{{2zk_{e} \delta _{n} a(t) - 2\chi _{n} P_{{M^{{at}} }}^{n} \left[ {E_{{nt}}^{i} (t)} \right]^{2} }} \\ & \chi _{n} ,i = 1,2,3,4. \\ \end{aligned} $$

According to Sect. 3.1, the social cost of carbon in industry, construction, transportation and power industry in economic Region $n$ is:

$$ \begin{aligned} SCC_{{nt}} (i) & = \frac{{2\chi _{n} P_{{M^{{at}} }}^{n} a(t)\overline{{Y_{n} }} \left[ {zk_{e} - 2E_{{nt}}^{i} (t)} \right]}}{{zk_{e} \left[ {2\delta _{n} a(t) - \chi _{n} P_{{M^{{at}} }}^{n} E_{{nt}}^{i} (t)} \right]}}, \\ & i = 1,2,3,4. \\ \end{aligned} $$

Among them, 1,2,3,4 respectively represent industry, construction, transportation, and power. If the emission control rate reaches the upper limit value of 1, the optimal carbon tax for the industrial, construction, transportation, and power industries in the economic Region $n$ is,

$$ \begin{aligned} \tau _{{nt}}^{*} (i) & = \frac{{2\chi _{n} P_{{M^{{at}} }}^{n} a(t)\overline{{Y_{n} }} \left[ {zk_{e} - 2E_{{ni}}^{i} (t)} \right] - 2a(t)\overline{{Y_{n} }} zk_{e} \chi _{n} \lambda _{{ni}} }}{{zk_{e} \left[ {2\delta _{n} a(t) - \chi _{n} P_{{M^{{at}} }}^{n} E_{{ni}}^{i} (t)} \right] + zk_{e} \chi _{n} \lambda _{{ni}} }}, \\ & i = 1,2,3,4. \\ \end{aligned} $$

### Optimal emission control rate, social cost of carbon, optimal consumption

The optimal emission control rate and social cost of carbon of key industries in the economic Region $n$ is,

$$ \begin{aligned} \mu _{{ni}} & = \frac{{zk_{e} \left( {P_{{M^{{at}} }}^{n} E_{{nt}}^{i} (t) - \lambda _{{ni}} } \right) - 2P_{{M^{{at}} }}^{n} \left[ {E_{{nt}}^{i} (t)} \right]^{2} }}{{2zk_{e} \delta _{n} a(t) - 2\chi _{n} P_{{M^{{at}} }}^{n} \left[ {E_{{nt}}^{i} (t)} \right]^{2} }}\chi _{n} , \\ & i = 1,2,3,4. \\ \end{aligned} $$

33 $$ \begin{aligned} & SCC_{{nt}} (i) = \frac{{2\chi _{n} P_{{M^{{at}} }}^{n} a(t)\overline{{Y_{n} }} \left[ {zk_{e} - 2E_{{nt}}^{i} (t)} \right]}}{{zk_{e} \left[ {2\delta _{n} a(t) - \chi _{n} P_{{M^{{at}} }}^{n} E_{{nt}}^{i} (t)} \right]}} \\ & = \frac{{2\chi _{n} N_{{1,1}} \varepsilon _{1} \eta _{1} \sum\limits_{{l = 1}}^{N} {\beta _{l}^{n} } \xi _{l} a(t)\overline{{Y_{n} }} \left[ {zk_{e} - 2E_{{nt}}^{i} (t)} \right]}}{{zk_{e} \left[ {2\delta _{n} a(t)M_{*}^{{at}} \left( {\delta _{n} + \varepsilon _{2} + \varphi _{{21}} - \frac{{\varphi _{{12}} \varphi _{{21}} }}{{\delta _{n} + \varphi _{{12}} }}} \right) - \chi _{n} N_{{1,1}} \varepsilon _{1} \eta _{1} \sum\limits_{{l = 1}}^{N} {\beta _{l}^{n} } \xi _{l} E_{{nt}}^{i} (t)} \right]}}, \\ & i = 1,2,3,4. \\ \end{aligned} $$

Among them, $i=1,2,3,4$ represents industry, construction, transportation, and power. The formula of social cost of carbon in the industries of industry, construction, transportation and power in the economic Region $n$ shows that the social cost of carbon increases with the increase of GNP. But the social cost of carbon is not linearly related to the emission control rates corresponding to industry, constructions, transportation and power.

The optimal consumption rate $\chi _{n}^{*}=\frac{{{C_n}}}{{{Y_n}}}$ satisfies the Linear algebra Eq. $\beta _{n}^{n}{A_n} \cdot \chi _{n}^{*}={\delta _n},$

34 $$\chi _{n}^{*}=\frac{{{\delta _n}}}{{\beta _{n}^{n}{A_n}}}.$$

Equation (44) shows that the consumption rate $\chi _{n}^{*}$ is positively correlated with the Bank rate ${\delta _n}$. The larger the ${\delta _n}$, the greater the focus on current consumption, with investment and emissions reducing and total consumption increasing. Consumption rate $\chi _{n}^{*}$ is negatively correlated with Total-factor productivity ${A_n}$ and $\beta _{n}^{n}$.

## SCC and comparison of cooperative games between economic regions

### Social cost of carbon under the optimal emission reduction strategy in the economic region

The welfare function of economic Region $n$ under consumption reduction strategy $\pi$ in Sect. 3.2 is ${J_n}\left( \pi \right)$. Suppose there is a social planner who chooses a nationally optimal consumption reduction strategy to maximize the social welfare function. The welfare function of social planners under consumption reduction strategy $\pi$ is denoted as $\widetilde {J}\left( \pi \right)$. The welfare function is represented by the weighted sum of the welfare of each economic Region as $\widetilde {J}\left( \pi \right)=\sum\nolimits_{{n=1}}^{N} {{\vartheta _{nt}}{J_n}\left( \pi \right)}$. Where ${\vartheta _{nt}}$ represents the weight of the economic Region in the welfare function of social planners, which meets $\sum\nolimits_{{n=1}}^{N} {{\vartheta _{nt}}=} 1,{\vartheta _{nt}} \geqslant 0,\forall t>0.$

Under a set of utility weights, the consumption reduction strategy for social planners to achieve maximum welfare is recorded as $${\widehat {\pi }_n}=\left( {\widehat {C}_{1}^{n}, \ldots ,\widehat {C}_{N}^{n},{{\widehat {\mu }}_n}} \right),n=1, \ldots ,N.$$ This is the optimal consumption reduction strategy. For any consumption reduction strategy $${\pi _n}=\left( {C_{1}^{n}, \ldots ,C_{N}^{n},{\mu _n}} \right),$$$$n=1, \ldots ,N,$$ there is $$\widetilde {J}\left( \pi \right) \leqslant \widetilde {J}\left( {\widehat {\pi }} \right).$$.

Social planners achieve maximum social welfare (value function), that is, under all consumption reduction strategies, there exists one consumption reduction strategy that maximizes social welfare under that strategy, which can be expressed mathematically as $\widetilde {J}=\mathop {\hbox{max} }\limits_{\pi } \left\{ {\widetilde {J}\left( \pi \right)} \right\}.$ The value function of economic Region $n$ is denoted as ${\tilde {J}^n}$ by social planners and satisfies:

35 $$\tilde {J}=\sum\limits_{{n=1}}^{N} {{\vartheta _n}{{\tilde {J}}^n}} .$$

We define the optimal trajectory of the state variable generated by the solution of the social planner as $\left( {\widehat {M},\widehat {T},\widehat {K}} \right).$.

#### Assumption

The subjective Bank rate ${\delta _n}$ of economic Region $n$ is the same. The HJB equation for social planners is:

36 $${\delta _n}\tilde {J}=\mathop {\hbox{max} }\limits_{{\chi _{1}^{n}, \cdots ,\chi _{N}^{n},\mu _{{n(i=1,2,3,4)}}^{i}}} \left\{ \begin{gathered} \frac{{\partial \tilde {J}}}{{\partial t}}+{\delta _n}\sum\limits_{{l=1}}^{N} {{\vartheta _l}} \log \left( {\gamma {C_{nt}}+(1 - \gamma )carb} \right) \hfill \\ +\sum\limits_{{l=1}}^{N} {\frac{{\partial \tilde {J}}}{{\partial {K_l}}}\frac{{\partial {K_l}}}{{\partial t}}+\frac{{\partial \tilde {J}}}{{\partial {M^{at}}}}\frac{{\partial {M^{at}}}}{{\partial t}}+\frac{{\partial \tilde {J}}}{{\partial {M^{uo}}}}\frac{{\partial {M^{uo}}}}{{\partial t}}} \hfill \\ +\frac{{\partial \tilde {J}}}{{\partial {M^{lo}}}}\frac{{\partial {M^{lo}}}}{{\partial t}}+\frac{{\partial \tilde {J}}}{{\partial {T^{at}}}}\frac{{\partial {T^{at}}}}{{\partial t}}+\frac{{\partial \tilde {J}}}{{\partial {T^o}}}\frac{{\partial {T^o}}}{{\partial t}} \hfill \\ \end{gathered} \right\}.$$

As in Sect. 3.2, the HJB of the social planner is rewritten as:

37 $$ \begin{aligned} \delta _{n} \tilde{J} = & \mathop {\max }\limits_{{\chi _{1}^{n} , \cdots ,\chi _{N}^{n} ,\mu _{{n(i = 1,2,3,4)}}^{i} }} \\ & \times \left\{ \begin{gathered} \frac{{\partial \tilde{J}}}{{\partial t}} + \delta _{n} \sum\limits_{{l = 1}}^{N} {\vartheta _{l} } \sum\limits_{{l = 1}}^{N} {\beta _{l}^{n} \log \left( {\left( {\gamma + (1 - \gamma )\omega } \right)\chi _{l}^{n} A_{l} K_{l} } \right)} \hfill \\ + \sum\limits_{{l = 1}}^{N} {\frac{{\partial \tilde{J}}}{{\partial K_{l} }}K_{l} \left[ {g_{l} (t,\chi _{l} ,\mu _{{nl}} ) - \xi _{l} T_{t}^{{at}} } \right]} \hfill \\ + \frac{{\partial \tilde{J}}}{{\partial T^{{at}} }}\left( {\varepsilon _{1} \left[ {\eta _{0} + \eta _{1} \frac{{M_{t}^{{at}} }}{{M_{*}^{{at}} }} + F^{{ex}} (t)} \right] - \left( {\varepsilon _{2} + \varphi _{{21}} } \right)T^{{at}} + \varphi _{{21}} T^{o} } \right) \hfill \\ + \frac{{\partial \tilde{J}}}{{\partial T^{o} }}\left( {\varphi _{{12}} T^{{at}} - \varphi _{{12}} T^{o} } \right) + \frac{{\partial \tilde{J}}}{{\partial M^{{lo}} }}\left( {\phi _{{23}} M^{{uo}} - \phi _{{32}} M^{{lo}} } \right) \hfill \\ + \frac{{\partial \tilde{J}}}{{\partial M^{{at}} }}\left( { - \phi _{{12}} M^{{at}} + \phi _{{21}} M^{{uo}} + \sum\limits_{{l = 1}}^{N} {E_{l} \left( {1 - \frac{{\sum\limits_{{l = 1}}^{N} {E_{l} } }}{{zk_{e} }}} \right)} } \right) \hfill \\ + \frac{{\partial \tilde{J}}}{{\partial M^{{uo}} }}\left( {\phi _{{12}} M^{{at}} - \left( {\phi _{{21}} + \phi _{{23}} } \right)M^{{uo}} + \phi _{{32}} M^{{lo}} } \right) \hfill \\ \end{gathered} \right\}. \\ \end{aligned} $$

According to Sect. 3.2, the value function of the economic Region $n$ for social planners is:

38 $$\begin{gathered} \tilde {J}(t,M,T,K)=\sum\limits_{{l=1}}^{N} {\beta _{l}^{n}\log ({K_l}) - P_{{{M^{at}}}}^{n}{M^{at}}} - P_{{{M^{uo}}}}^{n}{M^{uo}} - P_{{{M^{lo}}}}^{n}{M^{lo}} \hfill \\ - P_{{{T^{at}}}}^{n}{T^{at}} - P_{{{T^o}}}^{n}{T^o}+{p^n}(t). \hfill \\ \end{gathered}$$

Substituting into Eq. (39),so that${K_l}=1,\left( {l=1, \ldots ,N} \right)$, we have,

39 $$ \begin{aligned} 0 = & \mathop {\max }\limits_{{C_{1}^{n} , \cdots ,C_{N}^{n} ,\mu _{n}^{1} , \cdots ,\mu _{n}^{4} }} \\ & \times \left\{ \begin{gathered} \sum\limits_{{l = 1}}^{N} {\vartheta _{l} } p_{t}^{{n,sp}} + \delta _{n} \sum\limits_{{l = 1}}^{N} {\vartheta _{l} } \sum\limits_{{l = 1}}^{N} {\beta _{l}^{n} \log \left( {\left( {\gamma + (1 - \gamma )\omega } \right)\chi _{l}^{n} A_{l} K_{l} } \right)} \hfill \\ + \sum\limits_{{l = 1}}^{N} {\vartheta _{l} } \sum\limits_{{l = 1}}^{N} {\beta _{l}^{n} \left[ {g_{l} (t,\chi _{l} ,\mu _{{nl}} ) - \xi _{l} T_{t}^{{at}} } \right]} \hfill \\ - \sum\limits_{{l = 1}}^{N} {\vartheta _{l} } P_{{T^{{at}} }}^{n} \left( {\varepsilon _{1} \left[ {\eta _{0} + \eta _{1} \frac{{M_{t}^{{at}} }}{{M_{*}^{{at}} }} + F^{{ex}} (t)} \right] - \left( {\varepsilon _{2} + \varphi _{{21}} } \right)T^{{at}} + \varphi _{{21}} T^{o} } \right) \hfill \\ - \sum\limits_{{l = 1}}^{N} {\vartheta _{l} } P_{{T^{o} }}^{n} \left( {\varphi _{{12}} T^{{at}} - \varphi _{{12}} T^{o} } \right) - \sum\limits_{{l = 1}}^{N} {\vartheta _{l} } P_{{M^{{lo}} }}^{n} \left( {\phi _{{23}} M^{{uo}} - \phi _{{32}} M^{{lo}} } \right) \hfill \\ - \sum\limits_{{l = 1}}^{N} {\vartheta _{l} } P_{{M^{{at}} }}^{n} \left( { - \phi _{{12}} M^{{at}} + \phi _{{21}} M^{{uo}} + \sum\limits_{{l = 1}}^{N} {E_{l} \left( {1 - \frac{{\sum\limits_{{l = 1}}^{N} {E_{l} } }}{{zk_{e} }}} \right)} } \right) \hfill \\ - \sum\limits_{{l = 1}}^{N} {\vartheta _{l} } P_{{M^{{uo}} }}^{n} \left( {\phi _{{12}} M^{{at}} - \left( {\phi _{{21}} + \phi _{{23}} } \right)M^{{uo}} + \phi _{{32}} M^{{lo}} } \right) \hfill \\ + \delta _{n} \sum\limits_{{l = 1}}^{N} {\vartheta _{l} } \left( {P_{{M^{{at}} }}^{n} M^{{at}} + P_{{M^{{uo}} }}^{n} M^{{uo}} + P_{{M^{{lo}} }}^{n} M^{{lo}} + P_{{T^{{at}} }}^{n} T^{{at}} + P_{{T^{o} }}^{n} T^{o} - p^{{n,sp}} (t)} \right) \hfill \\ \end{gathered} \right\}. \\ \end{aligned} $$

Based on Sect. 3.2, which derives the first-order conditions for the emission control rates of industry, constructions, transportation, and power, as well as the first-order conditions for the consumption rate, we obtain the emission control rates for industry, constructions, transportation, and power as:

40 $${\mu _{ni}}{\mathrm{=}}\frac{{z{k_e}\left( {\sum\limits_{{l=1}}^{N} {{\vartheta _l}} P_{{{M^{at}}}}^{l}E_{{nt}}^{i}(t) - {\lambda _{ni}}} \right) - 2\sum\limits_{{l=1}}^{N} {{\vartheta _l}} P_{{{M^{at}}}}^{l}{{\left[ {E_{{nt}}^{i}(t)} \right]}^2}}}{{2z{k_e}{\delta _n}a(t) - 2{\chi _n}\sum\limits_{{l=1}}^{N} {{\vartheta _l}} P_{{{M^{at}}}}^{l}{{\left[ {E_{{nt}}^{i}(t)} \right]}^2}}}{\chi _n}.$$

The optimal proportion of consumption in the economic Region $\:n$ is:

41 $${\mathop \chi \limits^{ \wedge } _n}=\frac{{\delta {\vartheta _n}}}{{{A_n}\sum\limits_{{l=1}}^{N} {{\vartheta _l}\beta _{n}^{l}} }}.$$

The social cost of carbon of industry, constructions, transportation and power in the economic Region $n$ is given by the following formula:

42 $$SC{C_{nt}}(i)=\frac{{2{\chi _n}\sum\limits_{{l=1}}^{N} {{\vartheta _l}} P_{{{M^{at}}}}^{l}a(t)\overline {{{Y_n}}} \left[ {z{k_e} - 2E_{{nt}}^{i}(t)} \right]}}{{z{k_e}\left[ {2{\delta _n}a(t) - {\chi _n}\sum\limits_{{l=1}}^{N} {{\vartheta _l}} P_{{{M^{at}}}}^{l}E_{{nt}}^{i}(t)} \right]}},i=1,2,3,4.$$

The optimal carbon tax for industry, construction, transportation, and power in the economic Region $n$ is,

43 $$ \begin{aligned} \tau _{{nt}} (i) & = \frac{{2\chi _{n} \sum\limits_{{l = 1}}^{N} {\vartheta _{l} } P_{{M^{{at}} }}^{l} a(t)\overline{{Y_{n} }} \left[ {zk_{e} - 2E_{{nt}}^{i} (t)} \right] - 2a(t)\overline{{Y_{n} }} zk_{e} \chi _{n} \lambda _{{ni}} }}{{zk_{e} \left[ {2\delta _{n} a(t) - \chi _{n} \sum\limits_{{l = 1}}^{N} {\vartheta _{l} } P_{{M^{{at}} }}^{l} E_{{nt}}^{i} (t)} \right] + zk_{e} \chi _{n} \lambda _{{ni}} }}, \\ & i = 1,2,3,4. \\ \end{aligned} $$

#### Theorem 1

The carbon tax of industry, construction, transportation, and power industry in the economic Region $n$ is smaller than its social cost of carbon.

#### Proof

$$\begin{gathered} {\tau _{nt}}(i)=\frac{{2{\chi _n}\sum\limits_{{l=1}}^{N} {{\zeta _l}} P_{{{M^{at}}}}^{l}a(t)\overline {{{Y_n}}} \left[ {z{k_e} - 2E_{{nt}}^{i}(t)} \right] - 2a(t)\overline {{{Y_n}}} z{k_e}{\chi _n}{\lambda _{ni}}}}{{z{k_e}\left[ {2{\delta _n}a(t) - {\chi _n}\sum\limits_{{l=1}}^{N} {{\zeta _l}} P_{{{M^{at}}}}^{l}E_{{nt}}^{i}(t)} \right]+z{k_e}{\chi _n}{\lambda _{ni}}}} \hfill \\ <\frac{{2{\chi _n}\sum\limits_{{l=1}}^{N} {{\zeta _l}} P_{{{M^{at}}}}^{l}a(t)\overline {{{Y_n}}} \left[ {z{k_e} - 2E_{{nt}}^{i}(t)} \right]}}{{z{k_e}\left[ {2{\delta _n}a(t) - {\chi _n}\sum\limits_{{l=1}}^{N} {{\zeta _l}} P_{{{M^{at}}}}^{l}E_{{nt}}^{i}(t)} \right]}}=SC{C_{ni}}(t). \hfill \\ \end{gathered}$$

### Comparison with non-cooperative games theory

#### Theorem 2

The optimal consumption ratio under cooperative games is smaller than that under non cooperative games.

#### Proof

According to the formula derived earlier, the optimal consumption ratio in a cooperative game is.

${\mathop \chi \limits^{ \wedge } _n}=\frac{{\delta {\vartheta _n}}}{{{A_n}\sum\nolimits_{{l=1}}^{N} {{\vartheta _l}\beta _{n}^{l}} }}.$ The optimal consumption ratio under non-cooperative games is $\chi _{n}^{*}=\frac{\delta }{{\beta _{n}^{n}{A_n}}},$.

$${\mathop \chi \limits^{ \wedge } _n}=\frac{{\delta {\vartheta _n}}}{{{A_n}\sum\nolimits_{{l=1}}^{N} {{\vartheta _l}\beta _{n}^{l}} }}=\frac{\delta }{{{A_n}\left( {\beta _{n}^{n}+\sum\nolimits_{{l \ne n}} {\frac{{{\vartheta _l}}}{{{\vartheta _n}}}\beta _{n}^{l}} } \right)}}<\frac{\delta }{{{A_n}\beta _{n}^{n}}}=\chi _{n}^{*}.$$

#### Theorem 1

reveals that in a cooperative equilibrium, the carbon tax exceeds its theoretical social cost of carbon, a premium with profound institutional implications. It signifies the evolution of the carbon tax from a singular tool for correcting externalities into a composite policy lever that simultaneously fulfills financing and distributional functions. The excess portion essentially constitutes a fiscal transfer mechanism grounded in historical responsibility and payment capacity, designed to provide public funds for the climate club. These funds support the diffusion of emission-reduction technologies and adaptive investments in vulnerable regions. By embedding fairness principles within price signals, this design mitigates the benefit-cost asymmetry in cooperation, thereby enhancing the alliance’s incentive compatibility and political sustainability. It offers an operational policy paradigm for implementing transnational climate agreements.

The reduction in the optimal consumption rate under the cooperative game in Theorem 2 fundamentally represents participants’ intertemporal resource optimization to achieve Pareto improvements. Within the cooperative framework, regions internalize the negative externalities of carbon emissions through contractual agreements, thereby reallocating resources originally intended for current consumption toward emissions reduction investments and green capital accumulation. This resource transfer aims to avert greater welfare losses from future capital depreciation caused by climate shocks, embodying dynamic efficiency under cooperative strategies. Conversely, the Nash equilibrium in non-cooperative games fails to resolve free-riding issues. Individual rationality drives regional preferences toward immediate consumption, forming a collectively irrational high-carbon lock-in path that ultimately results in a suboptimal global welfare state.

## Numerical simulation and parametric analysis

In this paper, we study the social cost of carbon in eight major economic regions(See Table 1) in China through non-cooperative and cooperative games, and obtain the analytical solution of social cost of carbon based on specific assumptions.

### Numerical simulation

The numerical simulation is based on eight comprehensive economic Regions in China, and we consider the model with eight heterogeneous regions. Table 2 summarizes the regional composition of the eight economic Regions and the initial values of capital and output (trillion yuan) in 2015, etc. Among them, the capital stock in 2015 is calculated by referring to the method of the perpetual inventory method of Zhang Jun et al. [46]. This section for the baseline scenario, non-cooperative game scenario, cooperative game scenario to determine the optimal emission reduction strategy, and plot the resulting changes in the global average temperature, carbon dioxide concentration, GDP and carbon emissions of the evolution of the image.

**Table 2:** Regional range and initial values

Economic Region	Output	Consumption rate	Economic growth rate	Capital
Northeast Economic Region (Northeast E-Z)	5.777	0.38	0.017	19.914
Northern Coastal Economic Region (Northern C-E-Z)	13.236	0.51	0.055	37.935
Eastern Coastal Economic Region (Eastern C-E-Z)	13.813	0.43	0.081	32.908
Southern Coastal Economic Region (Southern C-E-Z)	10.250	0.41	0.090	23.618
Middle reaches of the Yellow River economic Region (M-R Yellow E-Z)	8.562	0.41	0.058	32.366
Middle reaches of the Yangtze River Economic Region (M-R Yangtze E-Z)	9.718	0.10	0.069	25.855
Southwest Economic Region (Southwest E-Z)	8.670	0.47	0.094	26.028
Northwest Economic Region (Northwest E-Z)	2.247	0.45	0.063	8.714

#### Baseline scenario

**Fig. 5:**
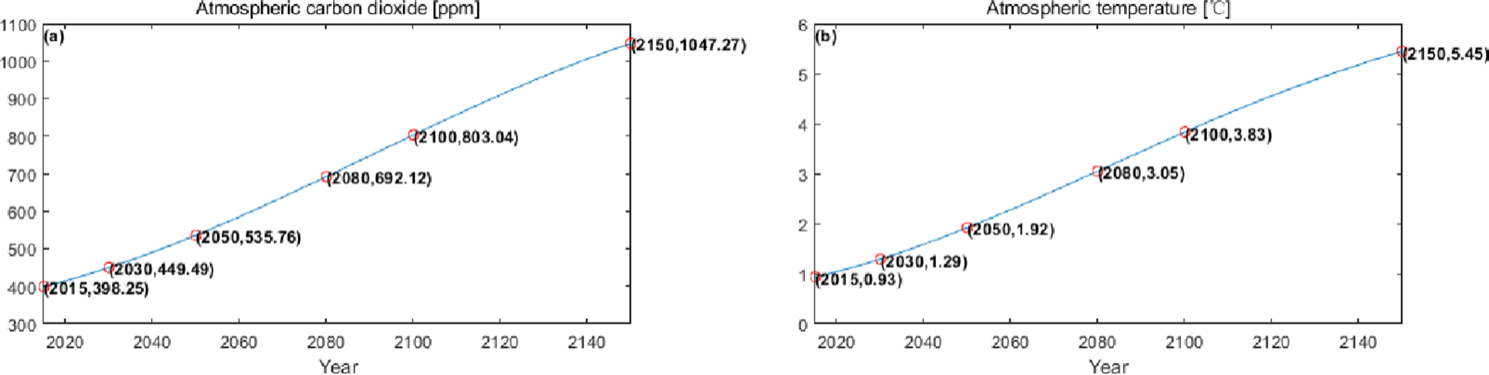
Atmospheric carbon dioxide concentration and temperature： (**a**) atmospheric carbon dioxide concentration (**b**) atmospheric temperature

**Fig. 6:**
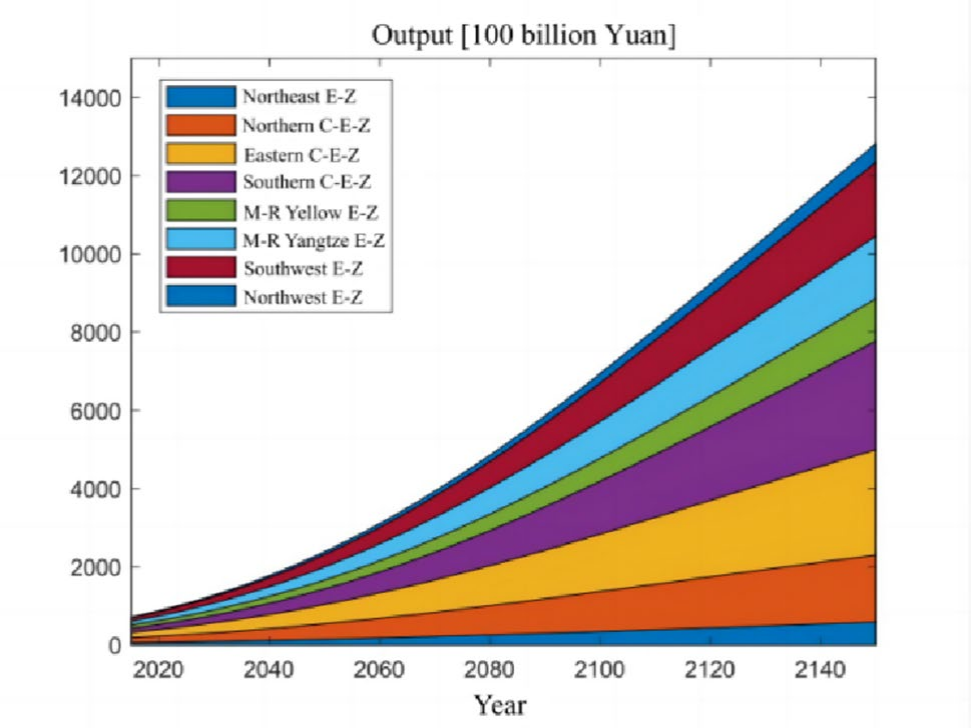
Baseline output

**Fig. 7:**
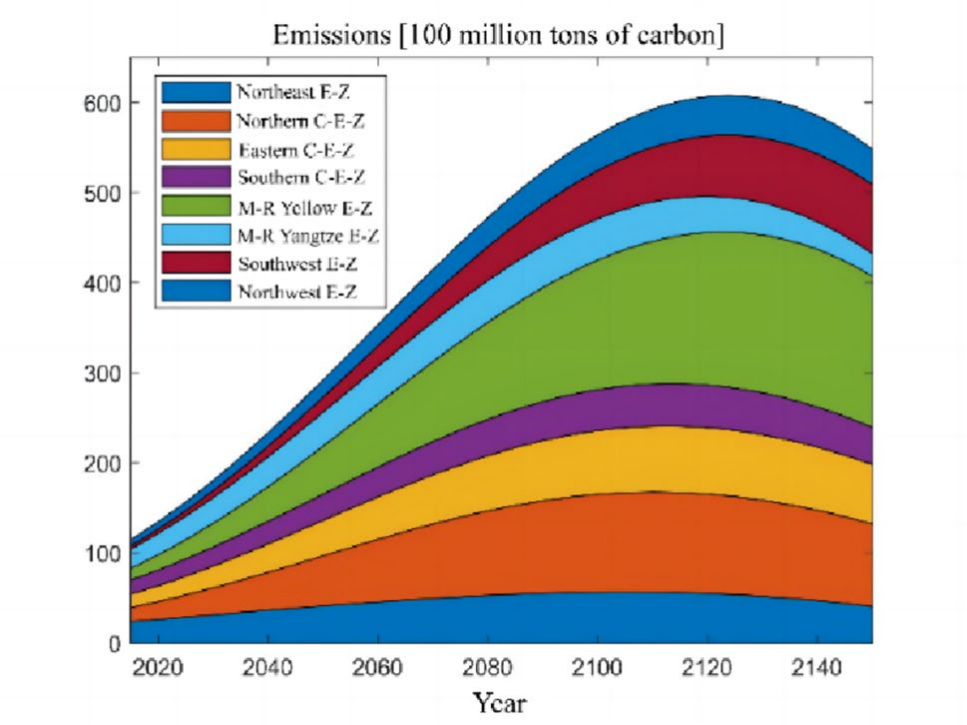
Baseline carbon emissions

Figure 5 shows the baseline evolution of atmospheric carbon dioxide concentration and atmospheric temperature. The ‘baseline’ scenario of this paper uses the standard DICE-2013R model and assumes that climate change policies have not changed since 2010. In the baseline scenario, the eight major economic Regions can only optimize their consumption and trade strategies. Figures 6 and 7 depict the changes in GDP and carbon emissions of the eight major economic Regions. The blue area at the bottom of the graph represents the Northeast Economic Region, followed by the Eastern Coastal Economic Region, Northern Coastal Economic Region, Southern Coastal Economic Region, Middle reaches of the Yellow River Economic Region, Middle reaches of the Yangtze River Economic Region, Southwest Economic Region, and Northwest Economic Region. In terms of GDP contribution rate, the eastern coastal economic Region has the highest, followed by the northern coastal economic Region and the southern coastal economic Region. In terms of carbon emission contribution rate, the economic Region in the middle reaches of the Yellow River has the highest, followed by the northern coastal economic Region and the eastern coastal economic Region. In the baseline scenario, our model predicts that by the end of this century, the national carbon dioxide emissions will be approximately 57.81 billion tons/year. These carbon emissions resulted in an atmospheric CO_2_ concentration of 803.04 ppm and an average temperature increase of approximately 3.83 ℃ at the end of this century.

Simulation results indicate that the SCC in the northern, eastern, and southern coastal economic zones is significantly higher than in inland regions. This phenomenon is an inevitable outcome of the combined effects of various drivers within the theoretical model presented in Sect. 3. According to the analytical solution for SCC (Eq. (43)), its value exhibits a positive correlation with regional output. As China’s economic engines, these coastal economic zones possess higher GDP levels and capital stocks. This translates to greater economic value flows per unit of carbon emissions, thereby amplifying the potential marginal capital damage from climate change. Given their massive economic scale, these regions perceive greater economic risk exposure per ton of carbon emissions, driving up their SCC valuations. Additionally, these areas typically exhibit higher total energy consumption and carbon emission baselines. In theoretical models, initial emission levels directly influence SCC by affecting the scale of damage internalization. Higher emission baselines imply a steeper marginal cost curve for emissions reductions, necessitating greater expenditures to meet global temperature targets. Simultaneously, these regions are more deeply embedded within domestic trade networks. As analyzed in Sect. 3.5, under heterogeneous damage parameters, a region’s SCC is the weighted sum of its own damage and the damage of other regions linked through trade. As national trade hubs, coastal economic zones incorporate substantial products from other economic zones into their consumption baskets. Consequently, they must internalize the climate damage their economic activities indirectly impose on the entire nation through supply chains by adopting higher SCCs.

#### Non-cooperative game scenarios

**Fig. 8:**
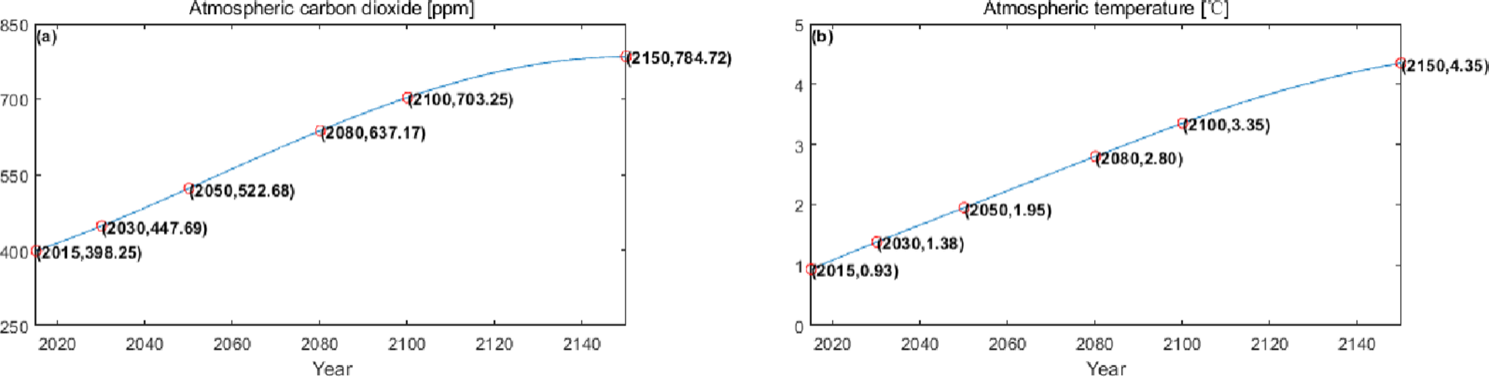
Atmospheric carbon dioxide concentration and atmospheric temperature under the non-cooperative game: (**a**) atmospheric carbon dioxide concentration (**b**) atmospheric temperature

**Fig. 9:**
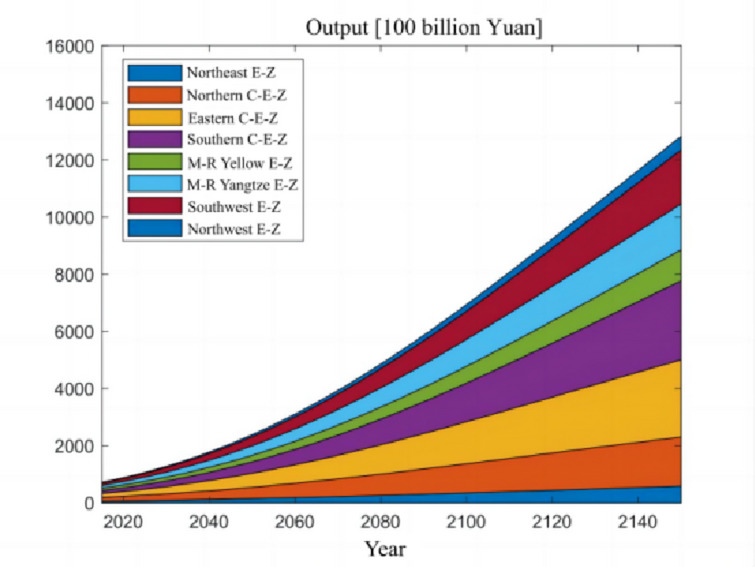
Output under non-cooperation

**Fig. 10:**
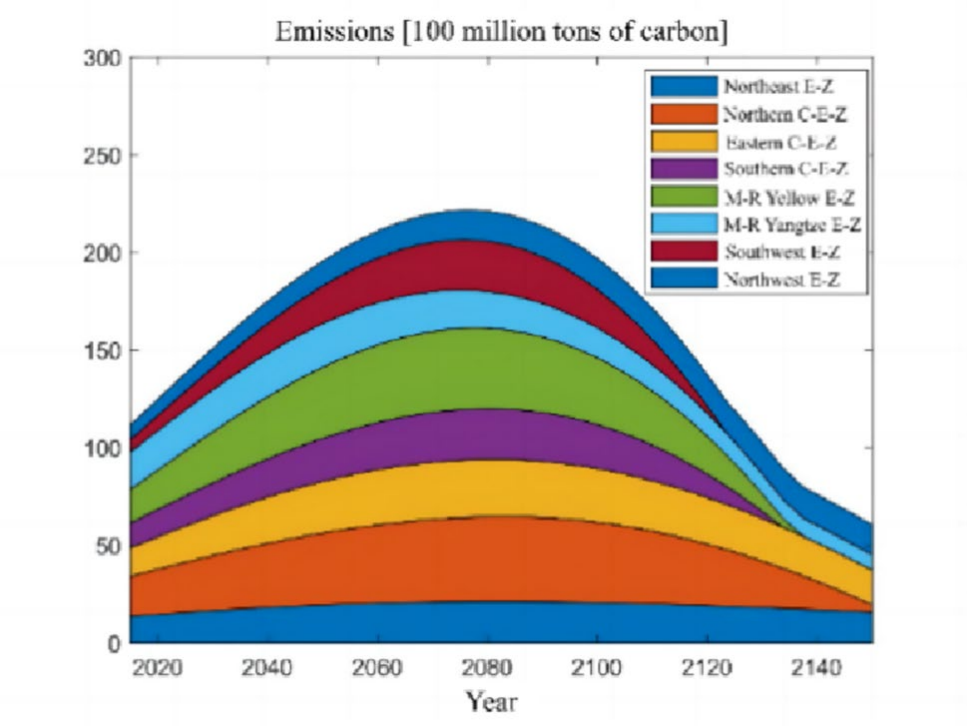
Carbon emissions under non-cooperation

Figure 8 depicts the evolution of the optimal control of atmospheric carbon dioxide concentration and average atmospheric temperature rise in a non-cooperative game scenario. Figures 9 and 10 depict the changes in gross domestic product and carbon emissions of the eight major economic regions. The blue area at the bottom of the graph represents the Northeast Economic Region, followed by the Eastern Coastal Economic Region, Northern Coastal Economic Region, Southern Coastal Economic Region, Middle reaches of the Yellow River Economic Region, Middle reaches of the Yangtze River Economic Region, Southwest Economic Region, and Northwest Economic Region. Compared with the baseline average atmospheric temperature increase of 3.83 ℃, the model in this paper indicates that the optimal control of emissions in this scenario resulted in a global average temperature increase of 3.35 ℃ by the end of this century. At the beginning of the next century, the concentration of carbon dioxide was 703.25 ppm.

**Fig. 11:**
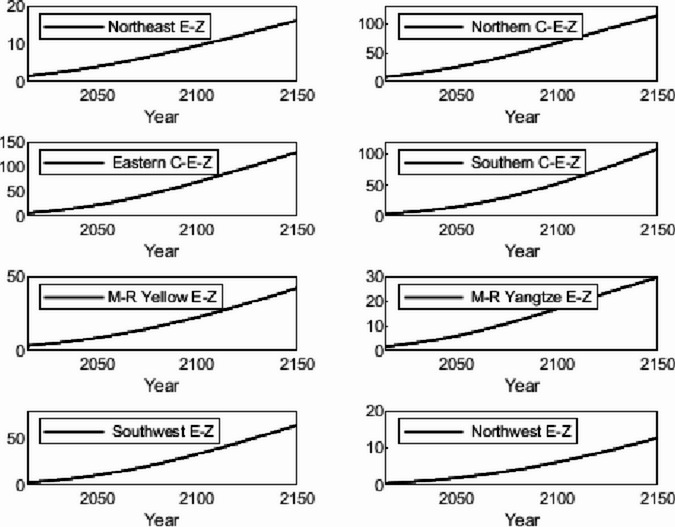
The social cost of industrial carbon under non-cooperation

**Fig. 12:**
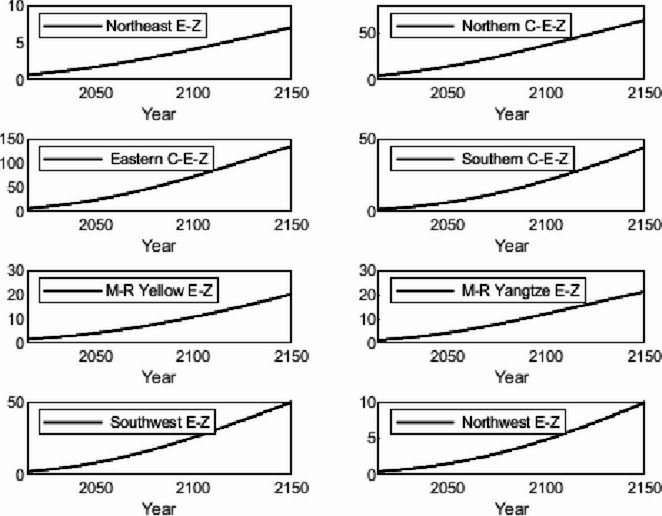
The social cost of constructions carbon under non-cooperation

**Fig. 13:**
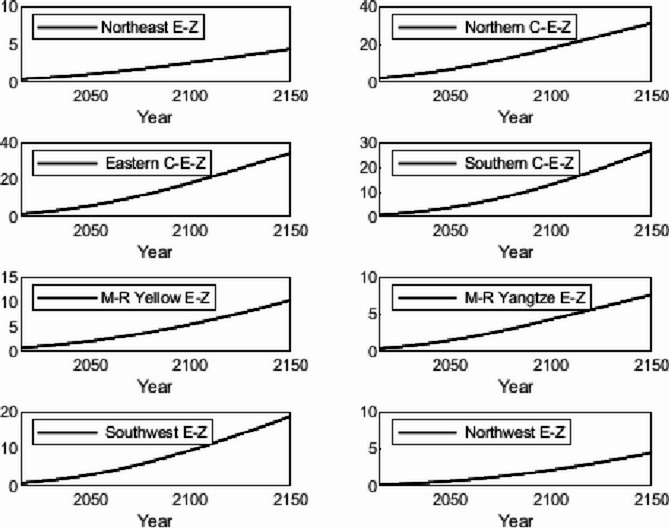
The social cost of transportation carbon under non-cooperation

**Fig. 14:**
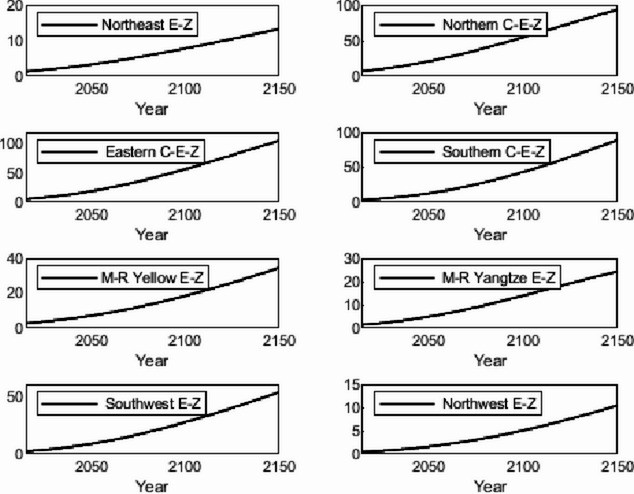
The social cost of power carbon under non-cooperation

Figures 11, 12, 13 and 14 draw the social cost of carbon images of the key industries of industry, construction, transportation and power in the eight economic Regions under the non-cooperative game.

#### Cooperative gaming scenarios

**Fig. 15:**
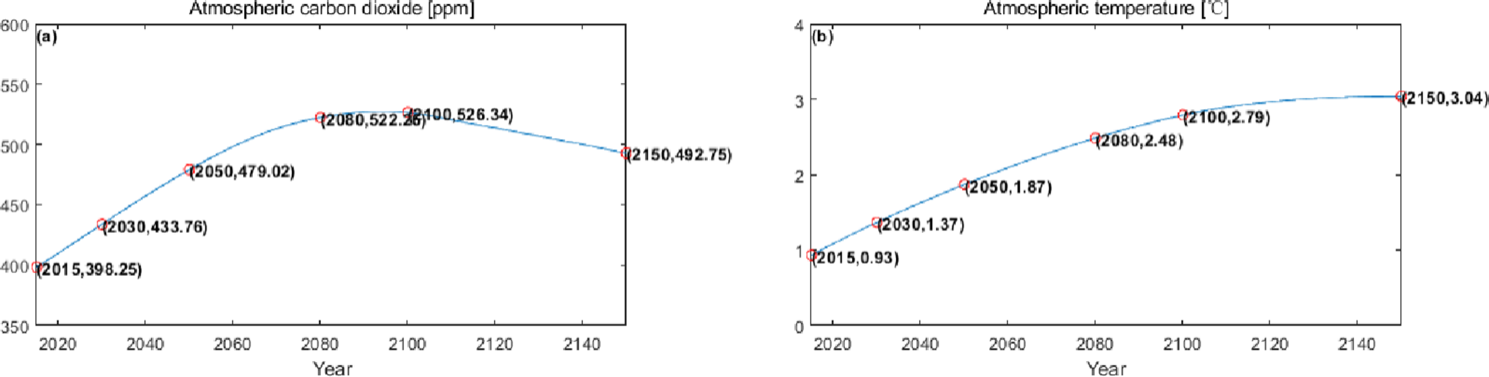
Atmospheric carbon dioxide concentration and atmospheric temperature under the cooperative game: (**a**) atmospheric carbon dioxide concentration (**b**) atmospheric temperature

**Fig. 16:**
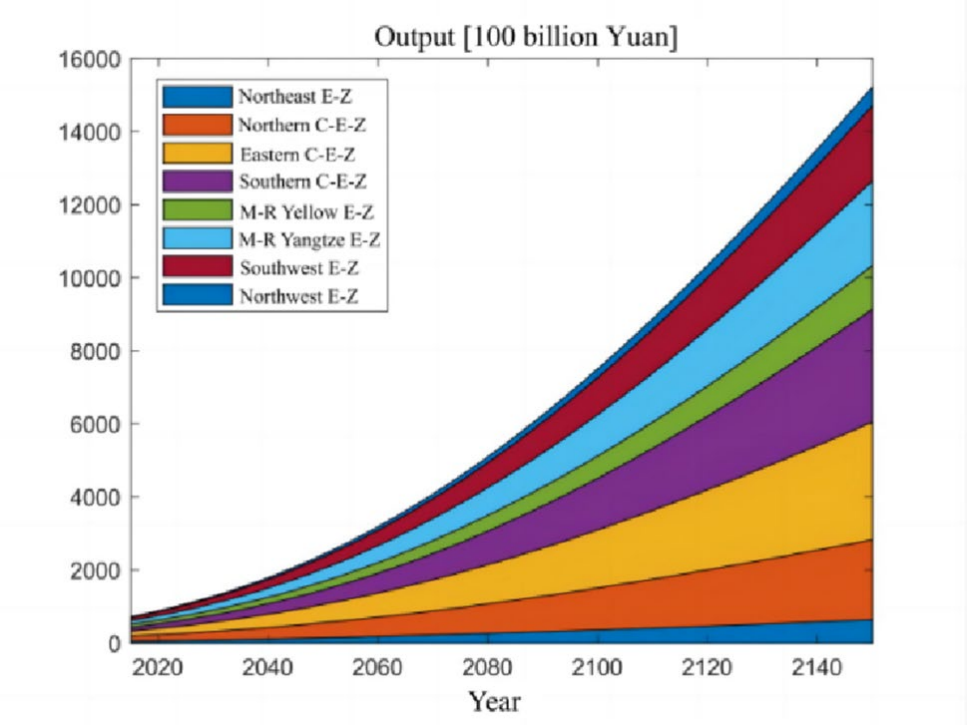
Output under the cooperative game

**Fig. 17:**
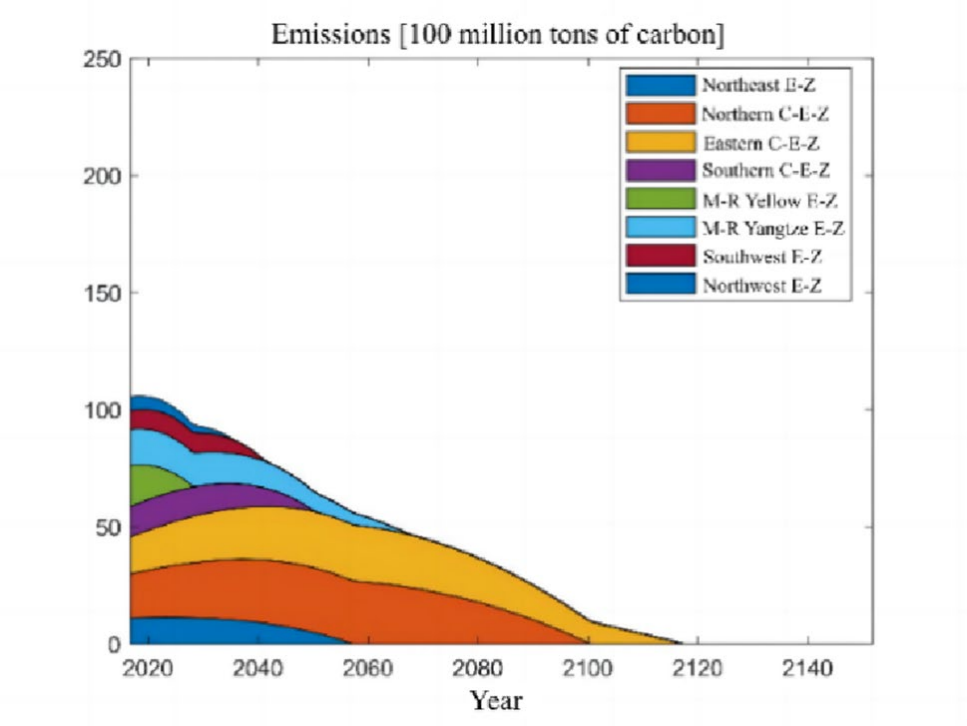
Carbon emissions under the cooperative game

Figure 15 depicts the evolution of the optimal control of atmospheric carbon dioxide concentration and average atmospheric temperature rise in a cooperative game scenario. Figures 16 and 17 depict the changes in GDP and carbon emissions of the eight major economic regions. The blue area at the bottom of the graph represents the Northeast Economic Region, followed by the Eastern Coastal Economic Region, Northern Coastal Economic Region, Southern Coastal Economic Region, Middle reaches of the Yellow River Economic Region, Middle reaches of the Yangtze River Economic Region, Southwest Economic Region, and Northwest Economic Region.

Figures 15, 16 indicate that under cooperative games, each economic Region adopts stricter behavioral strategies than under non-cooperative games. By implementing the optimal cooperative emission reduction strategy, the national carbon dioxide emissions are expected to reach a peak of 12.83 billion tons in 2031, and the carbon dioxide concentration is expected to reach a peak of 527.35 ppm in 2094. After 2094, the absorption capacity of the ocean layer would exceed anthropogenic emissions, resulting in a gradual decrease in atmospheric carbon dioxide concentration. By 2100, the carbon dioxide concentration is estimated to be 526.34ppm. Compared with the baseline and non-cooperative game scenarios, the carbon dioxide concentration would decrease by 34.46% and 25.16%. At the beginning of the next century, the average atmospheric temperature under cooperative games is predicted to increase to 2.79 ℃, which is 16.7% lower than the 3.35 ℃ increase under non-cooperative games. In cooperative game scenarios, lower ultimate temperature rise and earlier emissions peaking validate the core assertion of Theorem II. Cooperation reduces the globally optimal consumption rate, redirecting economic resources from short-term consumption toward long-term green investment. This manifests in the model as higher emission control rates and more proactive mitigation expenditures. Such collective action—investing in the future—effectively lowers the carbon intensity of the entire economic system and accelerates the green transformation of capital stock. Conversely, the free-riding incentives in non-cooperative scenarios lead to insufficient mitigation efforts, resulting in higher carbon emission trajectories and long-term climate damage, ultimately manifesting as higher societal carbon costs. This contrast powerfully demonstrates that the long-term benefits of interregional cooperation sufficiently offset the required short-term consumption sacrifices, providing a theoretical basis for establishing effective regional coordination mechanisms.

**Fig. 18:**
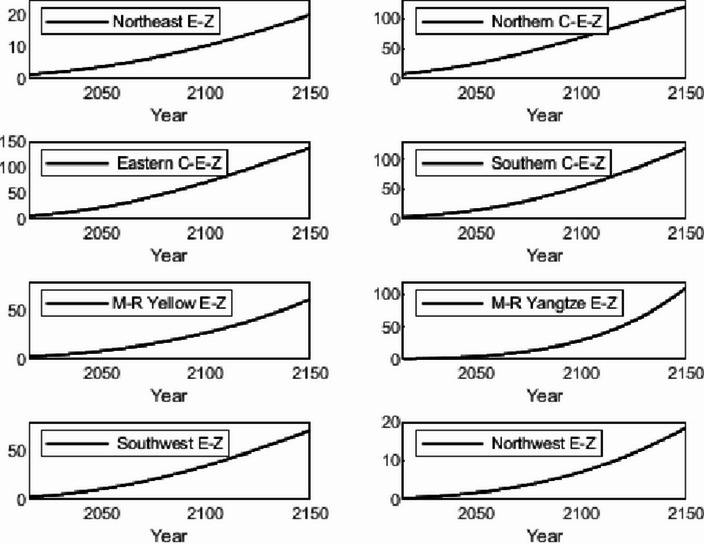
The social cost of industrial carbon under the cooperative game

**Fig. 19:**
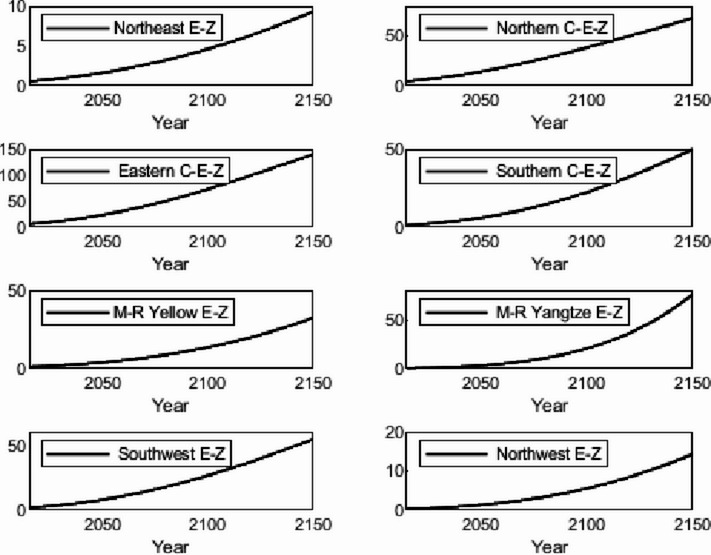
The social cost of constructions carbon under the cooperative game

**Fig. 20:**
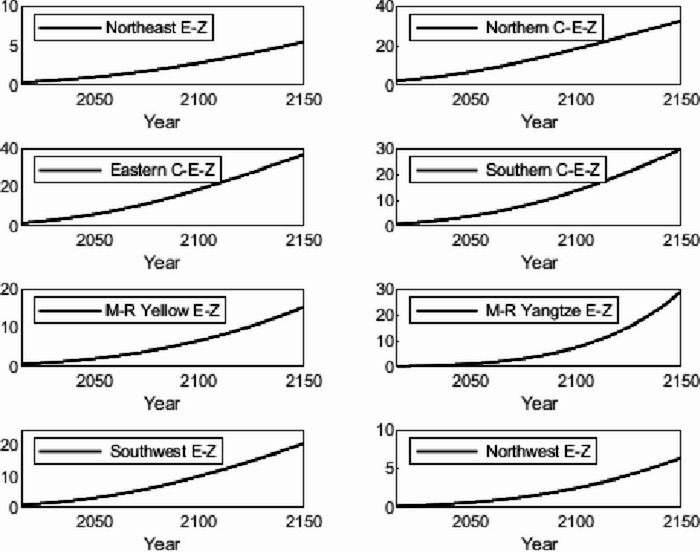
The social cost of transportation carbon under the cooperative game

**Fig. 21:**
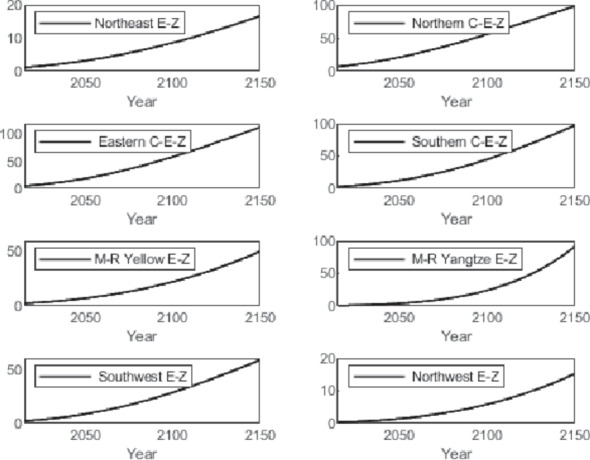
The social cost of power carbon under the cooperative game

Figures 18, 19, 20 and 21 draws the social cost of carbon image of industry, construction, transportation and power in the eight major economic Regions under the cooperation game.

The Sixth IPCC Report indicates that limiting warming to 1.5 °C (> 50%) or below 2 °C (> 67%) by the end of the century will require deep, rapid and sustained reductions in GHG emissions to reach net-zero emissions of CO_2_ and include significant reductions in emissions of other GHGs, in particular methane (high confidence). Various study scenarios in the Report indicate a more than 50 per cent probability of a global temperature rise of 1.5 °C or more between 2021 and 2040. Especially under a high emissions pathway, global temperatures could reach this tipping point even sooner (between 2018 and 2037); by 2100, global temperatures could rise between 3.3 °C and 5.7 °C, whereas the last time global temperatures exceeded pre-industrial levels by 2.5 °C was more than 3 million years ago. To limit global temperature rise to 1.5 °C and not exceed, or only marginally exceed, this range, greenhouse gas emissions would need to peak by 2025 at the latest, and then decline rapidly, by 43% by 2030 compared to 2019 levels, and by 60% by 2035. Under the 1.5 °C temperature rise target (which is not exceeded or only marginally exceeded) pathway, the world should achieve net zero CO_2_ emissions by mid-century. Therefore, this paper sequentially imposes a 1.5 °C versus 2 °C limit on the temperature module to explore regional heterogeneity under different temperature rise limits.

**Fig. 22:**
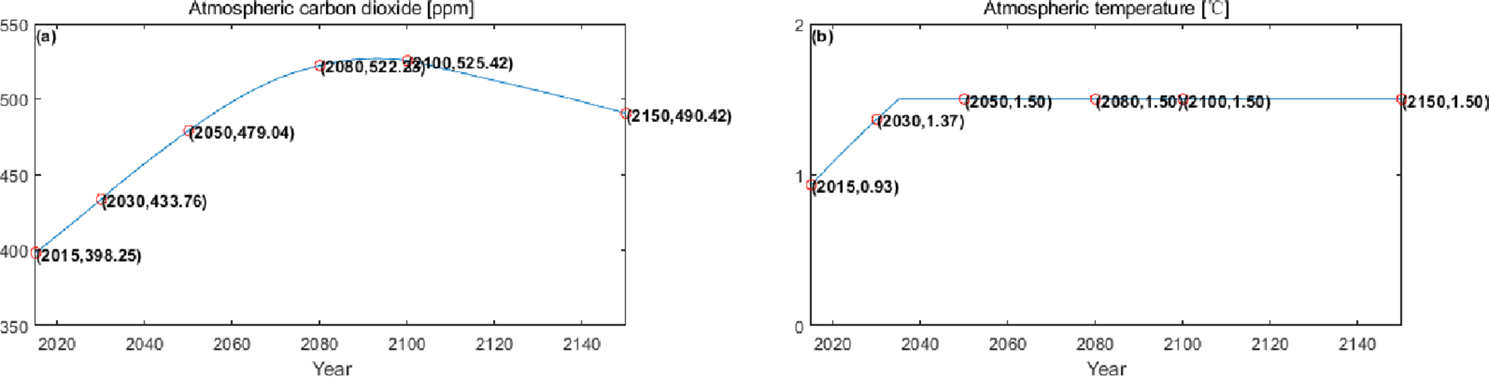
Atmospheric carbon dioxide concentration and atmospheric temperature at a 1.5 °C temperature rise: (**a**) atmospheric carbon dioxide concentration (**b**) atmospheric temperature

**Fig. 23:**
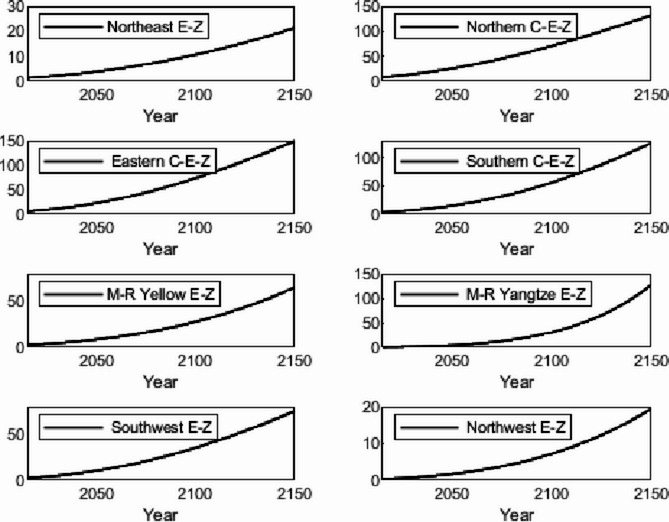
Social cost of industrial carbon by economic region for a 1.5°C temperature rise

**Fig. 24:**
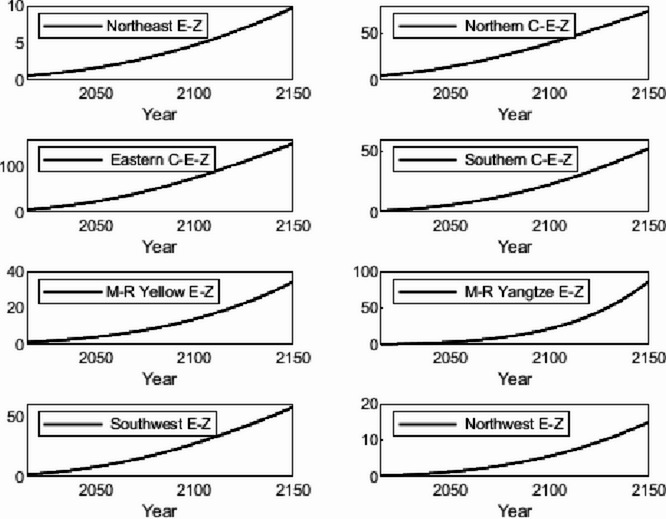
Social cost of carbon in constructions by economic Region for a 1.5°C temperature

**Fig. 25:**
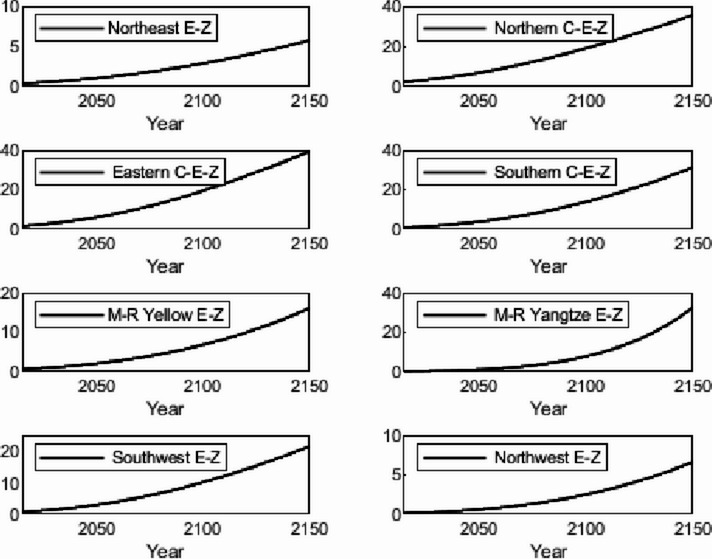
Social cost of transportation carbon by economic region at 1.5°C temperature rise

**Fig. 26:**
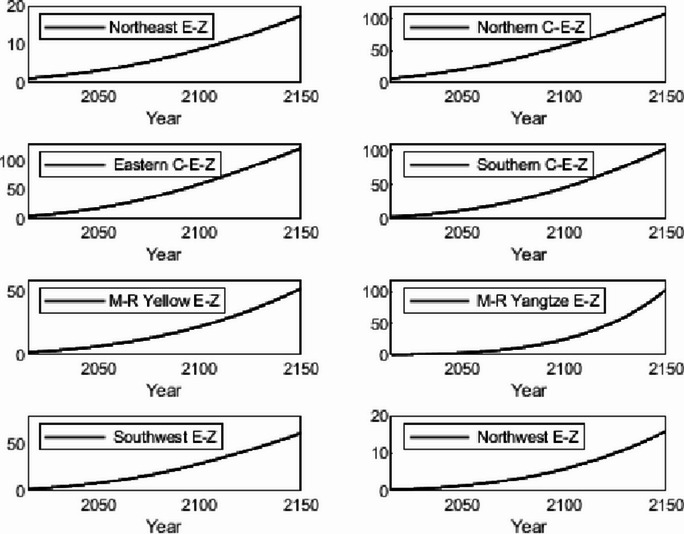
Social cost of carbon for power by economic region for a 1.5°C temperature rise

Figure 22 depicts the evolution of the optimal control of atmospheric CO_2_ concentration and mean atmospheric temperature rise for the atmospheric temperature limit of 1.5 °C in the cooperative game scenario. Figures 23, 24, 25 and 26 plot the images of the social cost of carbon for industry, constructions, transportation, and power in the key sectors of the eight economic regions under the 1.5 °C limit on temperature rise.

The simulation results in Fig. 22 show that the implementation of the 1.5 °C temperature rise constraint in the framework of the cooperative game will lead to a continuous climb in the national CO_2_ concentration, peaking at 921 ppm in 2150.In order to achieve this temperature control goal, carbon emissions must fall off a cliff while keeping economic output constant.

**Fig. 27:**
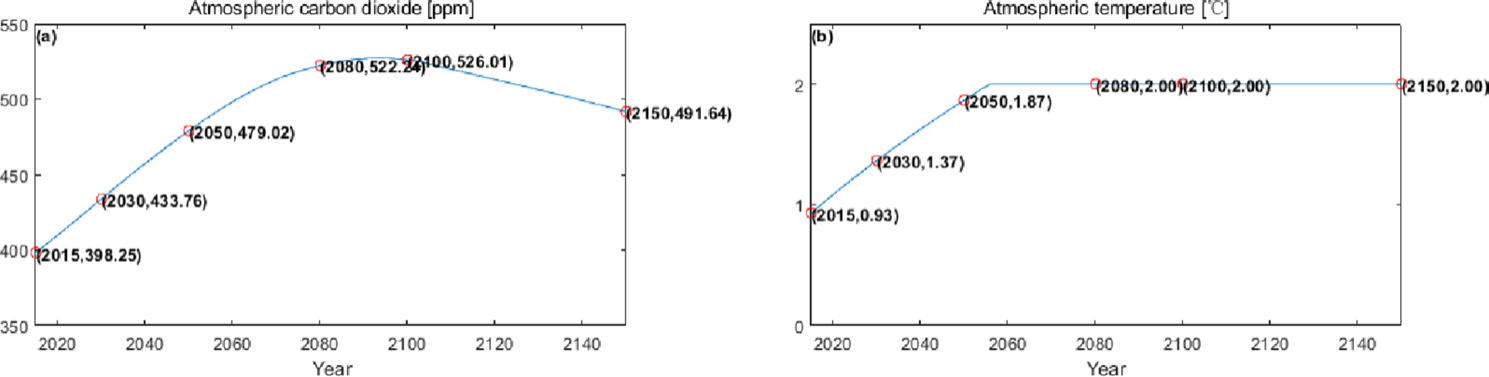
Atmospheric carbon dioxide concentration and atmospheric temperature at a 2 °C temperature rise: (**a**) atmospheric carbon dioxide concentration (**b**) atmospheric temperature

.

**Fig. 28:**
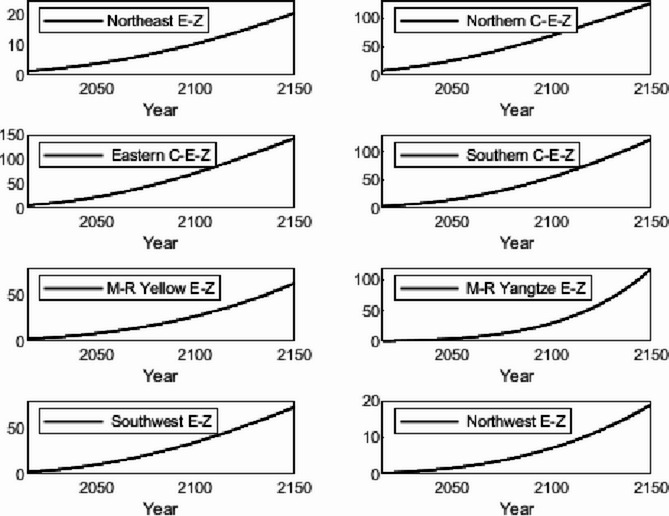
Social cost of industrial carbon by economic region for a 2 °C temperature rise

**Fig. 29:**
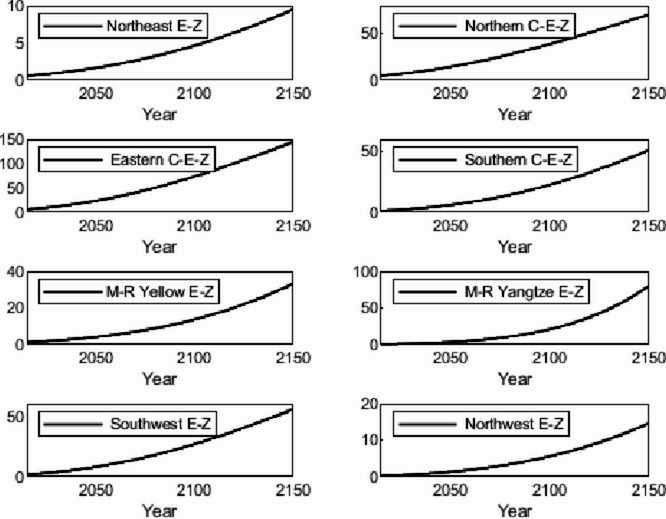
Social cost of carbon in constructions by economic Region for a 2 °C temperature

**Fig. 30:**
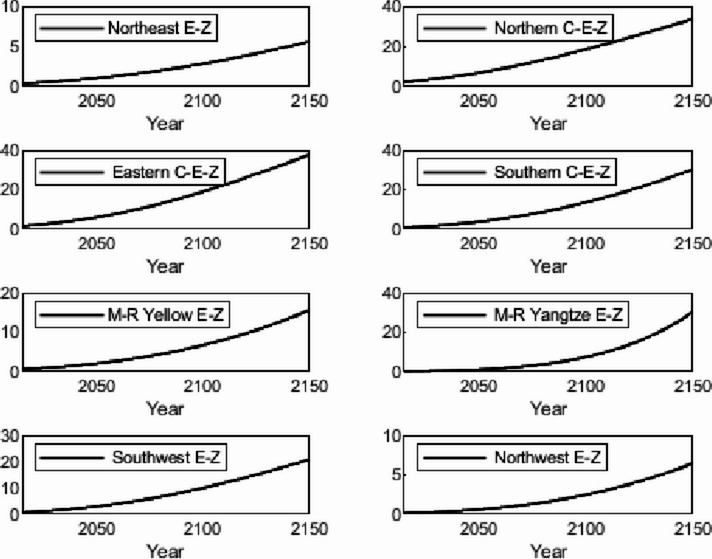
Social cost of transportation carbon by economic region at 2 °C temperature rise

**Fig. 31:**
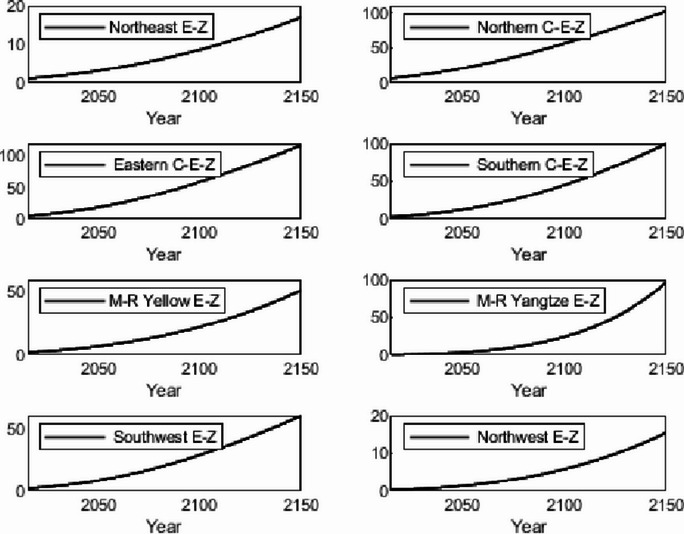
Social cost of carbon for power by economic region for a 2 °C temperature rise

**Fig. 32:**
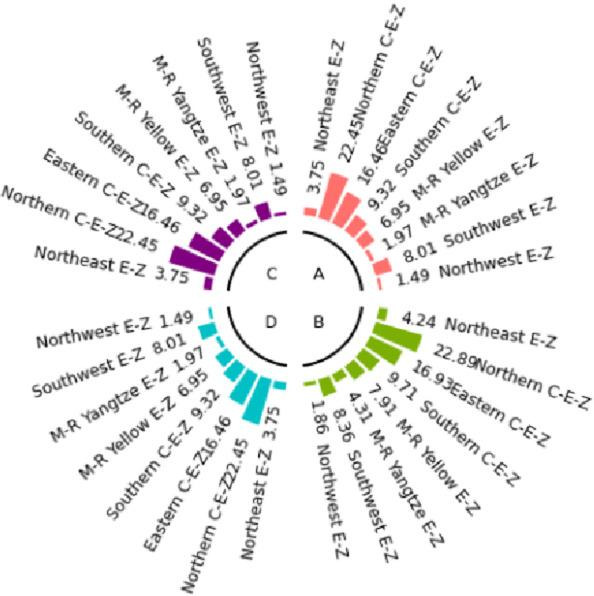
The social cost of carbon in 2015 for each economic region under four scenarios

**Fig. 33:**
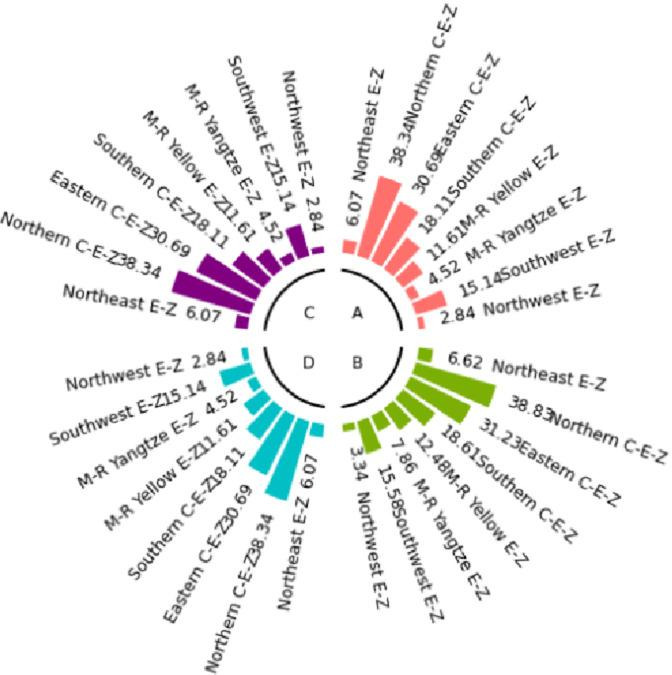
The social cost of carbon in 2030 for each economic region under four scenarios

**Fig. 34:**
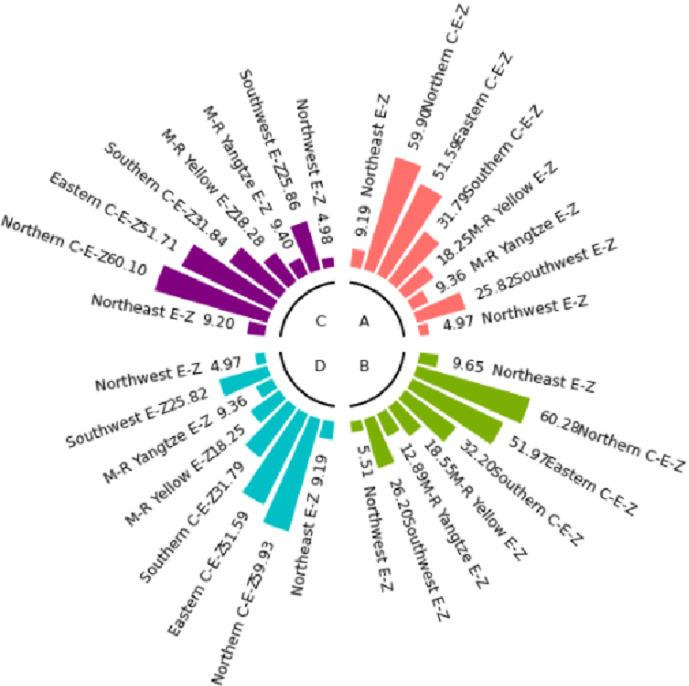
The social cost of carbon in 2045 for each economic region under four scenarios

**Fig. 35:**
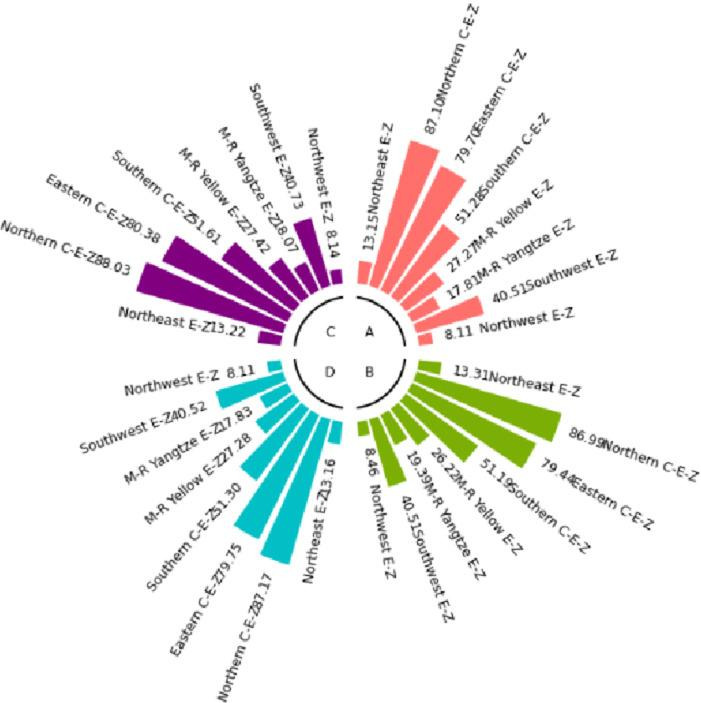
The social cost of carbon in 2060 for each economic region under four scenarios

**Fig. 36:**
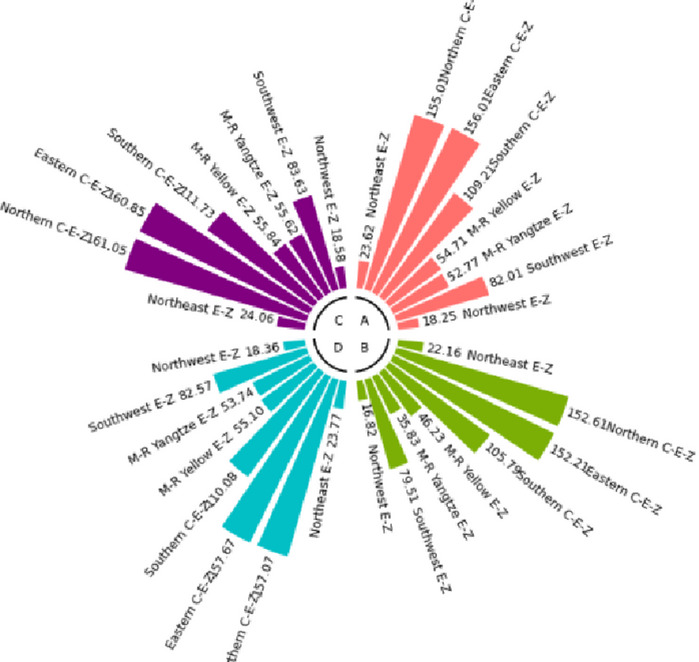
The social cost of carbon in 2090 for each economic region under four scenarios

**Fig. 37:**
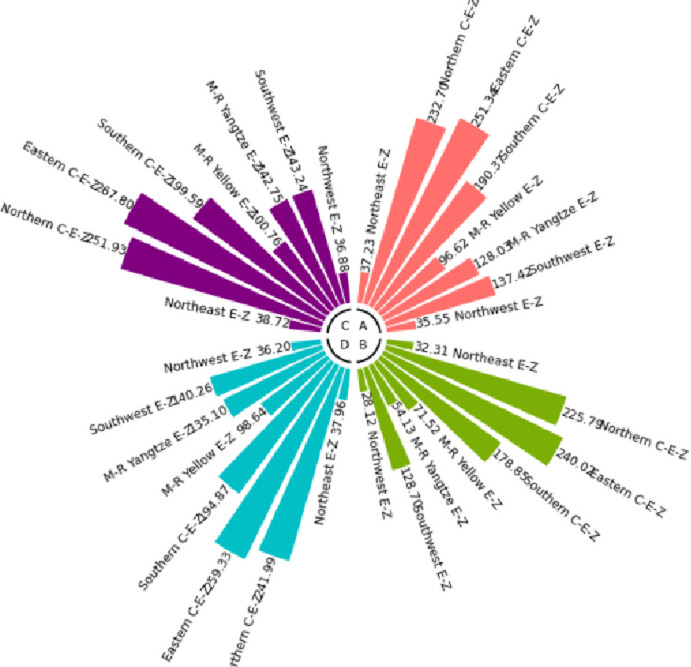
The social cost of carbon in 2120 for each economic region under four scenarios

**Fig. 38:**
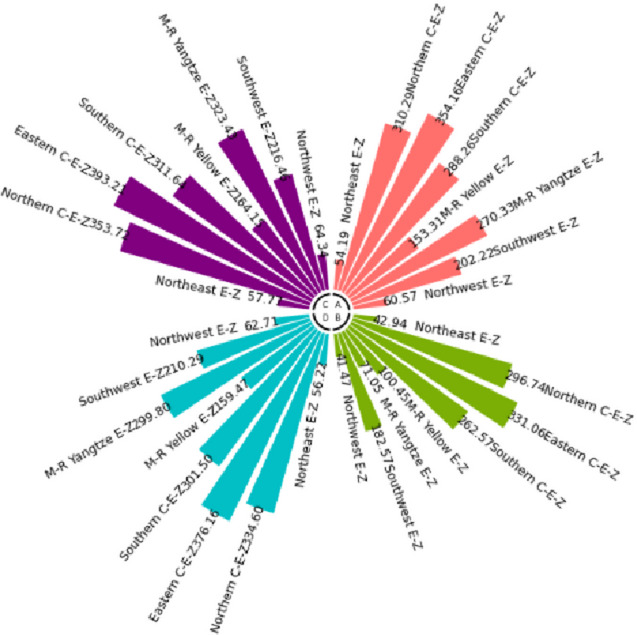
The social cost of carbon in 2150 for each economic region under four scenarios

Figure 27 depicts the evolution of the optimal control of atmospheric CO_2_ concentration and mean atmospheric temperature rise for the atmospheric temperature limit of 2 °C in the cooperative game scenario. Figures 28, 29, 30 and 31 plot images of the social cost of carbon for industry, constructions, transportation, and power for key industries in the eight economic regions under the 2 °C temperature rise limit. Figures 32, 33, 34, 35, 36, 37 and 38 plot the images of carbon social cost expressed in $/CO_2_ at the limited time nodes of each economic region under four scenarios of cooperative game, non-cooperative game, temperature rise of 1.5 °C, and temperature rise of 2 °C, respectively.

The simulation results in Fig. 27 show that the implementation of the 2 °C temperature rise constraint in the cooperative game framework will lead to a continuous climb in the national CO_2_ concentration, peaking at 914 ppm in 2150, which is slightly smaller than the scenario with the implementation of the 1.5 °C temperature rise constraint. To achieve this temperature-control goal, carbon emissions would need to undergo the same precipitous decline while holding economic output constant. Therefore, meeting the 1.5 °C (> 50%) or less than 2 °C (> 67%) warming constraint by the end of the century will require both technological advances that lead to an increase in the rate of control of emissions, and the adoption of cleaner energy sources that reduce carbon emissions.

In all scenarios, the SCC for the industrial and power sectors consistently exceeds that of the building and transportation sectors. This hierarchical difference can be directly traced to the model’s structural assumptions. As shown in Eq. (21), the sectoral SCC exhibits a negative correlation with its baseline carbon intensity and emission control rate. The industrial and power sectors possess significantly higher baseline carbon intensities and, due to their dominant share of national emissions, typically require stricter optimal emission control rates. The combination of high inherent carbon intensity and stringent emission reduction requirements pushes their marginal abatement costs—and consequently their SCC—to higher levels. In contrast, while equally critical, the building and transportation sectors exhibit relatively lower carbon intensity. Their emission reduction options in the short term may be more limited and costly, resulting in comparatively lower SCC. This finding indicates that climate policies must prioritize the electricity and industrial sectors, where the social cost of carbon is highest, to achieve cost-effective emissions reductions.

## Conclusions and policy implications

This paper improves a new integrated governance, environmental, and social model for regions and industries based on the ESG perspective. Under the model of this paper, it can be solved in closed form. To obtain a closed-form solution, the model introduces two key assumptions: the capital accumulation equation is a linear differential equation, and the immediate damage to output caused by temperature is temporarily disregarded in the production function. While these simplifications impose limitations, they preserve the core dynamic mechanisms. This paper derives analytical expressions for the social cost of carbon and optimal carbon tax for eight economic regions under cooperative and non-cooperative games on key industries: industry, construction, transportation, and electricity, as well as formulas for the regional social cost of carbon. The expressions of these solutions are easy to interpret and the effects of parameters on the solutions can be analyzed. The analytical formulas for the four major industries in the eight economic regions of China show that the social cost of carbon increases with the GNP, and the higher social cost of carbon corresponds to the higher emission control rate of the industry; the optimal consumption ratio under the cooperative game is lower than that under the non-cooperative game.

The findings of this paper have direct and indirect impacts on the policy formulation related to the early realization of carbon neutrality in China’s regional economic development and the policy formulation for the construction of regional ecological civilization. In particular, the following policy recommendations can be launched from the findings of this paper.

- China’s eight economic regions should increase cooperation in economic and social development. Based on the findings of this paper, cooperative games can significantly reduce the social cost of carbon, particularly by enhancing interregional trade cooperation. Only by increasing cooperation can the carbon social cost of the corresponding region be lower than the carbon social cost of non-cooperation.
- Given that numerical simulations indicate higher carbon social costs in the northern, eastern, and southern coastal economic zones compared to other regions, intensify the formulation and implementation of policies to reduce carbon emissions across sectors such as power generation, industry, construction, and transportation in each area. Additionally, implement stricter carbon pricing policies in regions with high SCC.
- Increase economic investment in carbon emission reduction. According to the model’s analysis of green capital investment and emission reduction expenditures, only by increasing economic investment in carbon emission reduction can green and low-carbon behaviors be implemented sooner.
- Encourage and guide industries and enterprises to continue pursuing technological innovation driven by green and low-carbon practices, particularly in the power and industrial sectors where carbon social costs are highest, to accelerate the emergence and industrialization of disruptive emission reduction technologies.
- Strengthen publicity and influence regarding green and low-carbon consumption behaviors among enterprises and residents. This aligns with the model’s setting of green consumption preferences, promoting voluntary, conscious, and self-disciplined adoption of green and low-carbon practices as the norm. This approach facilitates the emergence of optimal carbon emission rates and guides consumption toward optimal patterns.

## Appendix A

**Table:** 

Symbol	Meaning	Symbol	Meaning
$\:n,\:l$	Economic Zone Index	$\:t$	Time
$\:i$	Industry Index	$\:{\chi\:}_{n}$	Consumption Rate (Consumption to Output Ratio)
$\:{Y}_{nt}$	Total Output	$\:\omega\:$	Proportion of Emission Reduction-Related Consumption in Total Consumption
$\:{A}_{n}\left(t\right)$	Total Factor Productivity	$\:{A}_{n}^{ind}\left(t\right)$	Industrial Climate Change Mitigation Expenditure
$\:{K}_{nt}$	Capital Stock	$\:{A}_{n}^{arct}\left(t\right)$	Building Climate Change Mitigation Expenditure
$\:{L}_{nt}$	Labor Force	$\:{A}_{n}^{trans}\left(t\right)$	Transportation Climate Change Mitigation Expenditure
$\:{e}_{n}\left(t\right)$	Corporate Emission Reduction Investment Capital	$\:{A}_{n}^{pow}\left(t\right)$	Electric Power Climate Change Mitigation Expenditure
$\:{\alpha\:}_{n}$	Output Share of Capital	$\:{\delta\:}_{n}^{k}$	Capital Depreciation Rate
$\:{\beta\:}_{n}$	Output Share of Labor	$\:{\xi\:}_{n}$	Climate Damage Parameter
$\:{\mathcal{L}}_{n}^{1}\left(t\right)$	Basic Input Capital	$\:carb$	Emission Reduction Economic Input
$\:{\mathcal{L}}_{n}^{e}\left(t\right)$	Emission Reduction Economic Input Capital	$\:{E}_{nt}$	Regional Total Carbon Emissions
$\:{C}_{n}\left(t\right)$	Total Consumption	$\:{E}_{nt}^{ind}$	Industrial Carbon Emissions
$\:{c}_{n}\left(t\right)$	Per Capita Consumption	$\:{E}_{nt}^{arct}$	Building Carbon Emissions
$\:{a}_{n}\left(t\right)$	Emission Reduction Expenditure Function	$\:{E}_{nt}^{trans}$	Transportation Carbon Emissions
$\:{b}_{n}$	Convexity Parameter of Emission Reduction Cost	$\:{E}_{nt}^{pow}$	Electric Power Carbon Emissions
$\:{M}^{at}$	Atmospheric CO2 Content	$\:{E}_{nt}^{land}$	Land Use Carbon Emissions
$\:{M}^{uo}$	Surface Layer CO2 Content	$\:{\mu\:}_{ni}$	Sectoral Emission Control Rate
$\:{M}^{lo}$	Deep Ocean Layer CO2 Content	$\:{\sigma\:}_{n}^{i}\left(t\right)$	Carbon Intensity of Pre-mitigation Output
$\:{\varphi\:}_{ij}$	Carbon Diffusion Rate (i to j)	$\:{U}_{n}$	Instantaneous Utility of Economic Zone
$\:z$	CO2 Emission Evolution Coefficient	$\:{\eta\:}_{n}$	Elasticity of Marginal Utility of Consumption
$\:{k}_{e}$	Maximum Estimated CO2 Emissions	$\:{\delta\:}_{n}$	Subjective Time Discount Rate
$\:{T}^{at}$	Increase in Avg Atmospheric Temperature	$\:\gamma\:$	Consumption Portfolio Weight
$\:{T}^{o}$	Increase in Avg Ocean Temperature	$\:{C}_{l}^{n}$	Consumption of Economic Zone l’s Goods by Economic Zone n
$\:{\phi\:}_{ij}$	Thermal Diffusion Rate (i to j)	$\:{\beta\:}_{l}^{n}$	Consumption Preference Coefficient
$\:{\epsilon\:}_{1}$	Response Rate of Temp to Radiative Forcing	$\:{\rho\:}_{n}$	Utility Function Parameter
$\:{\epsilon\:}_{2}$	Rate of Atmos IR Radiation to Space	$\:{J}^{n}$	Value Function of Economic Zone n (Non-cooperative)
$\:{\epsilon\:}_{2}$	Total Radiative Forcing	$\:\stackrel{\sim}{J}$	Value Function of Social Planner (Cooperative)
$\:{\eta\:}_{0};{\eta\:}_{1}$	Climate Sensitivity Linear Parameter	$\:{\vartheta\:}_{nt}$	Weight of Economic Zone in Social Welfare Function
$\:{M}_{t}^{at}$	Pre-industrial CO2 Concentration	$\:{SCC}_{nt}$	Regional Social Cost of Carbon
$\:{F}^{ex}\left(t\right)$	Exogenous Radiative Forcing	$\:{SCC}_{nt}\left(i\right)$	Sectoral Social Cost of Carbon

## Appendix B

**Table:** 

Parameter Name	Parameter Value	Parameter Name	Parameter Value
Time Preference Rate $\delta$	0.03	Atmosphere to Shallow Ocean Transfer Coefficient ${\varphi _{12}}$	0.187
Capital Depreciation Rate ${\delta _k}$	0.08	Shallow Ocean to Atmosphere Transfer Coefficient ${\varphi _{21}}$	0.159
Atmospheric Carbon Stock ${M^{AT}}$	851GtC	Shallow Ocean to Deep Ocean Transfer Coefficient ${\varphi _{23}}$	0.067
Shallow Ocean Carbon Stock ${M^{UP}}$	460 GtC	Deep Ocean to Shallow Ocean Transfer Coefficient ${\varphi _{32}}$	0.0019
Deep Ocean Carbon Stock ${M^{LO}}$	1740 GtC	Atmosphere to Ocean Diffusion Coefficient ${\phi _{12}}$	0.44
Temperature Rise Damage Coefficient $\xi$	0.000176	Ocean to Atmosphere Diffusion Coefficient ${\phi _{21}}$	0.18
Radiative Forcing Coefficient $\eta$	3.8		

## Data Availability

Data will be made available on request.
